# Effectiveness and Persistence of Long‐Acting Injectable Cabotegravir and Rilpivirine in Migrant Individuals Living With HIV in Spain: Substudy of the RELATIVITY Cohort

**DOI:** 10.1002/jia2.70106

**Published:** 2026-04-21

**Authors:** Jara Llenas‐García, María del Mar Arcos Rueda, Ruth Calderón Hernaiz, Roberto Pedrero Tomé, Otilia Bisbal Pardo, Mariano Matarranz, Miguel Torralba, María José Galindo Puerto, Adrián Rodríguez, María Peñaranda Vera, Isabel Sanjoaquín Conde, Sara de la Fuente Moral, Alfonso Cabello‑Úbeda, Carolina Navarro San Francisco, Karenina Antelo Cuéllar, Marc Pedrosa Aragón, María Aguilera García, Juan Tiraboschi, Rosa María Martínez Álvarez, María Jesús Vivancos, Carmen Montero Hernández, Enrique Bernal Morell, Noemí Cabello‐Clotet, Luis Enrique Morano Amado, Laura Gisbert Pérez, María Antonia Sepúlveda, María Remedios Alemán Valls, Antonio Jesús Sánchez Guirao, Chiara Fanciulli, Cristina Escrig, Eva María Ferreira Pasos, Ana Lucas‐Dato, Sara García Torras, Carmen Hidalgo Tenorio, Miriam Estébanez, Magdalena Muelas‑Fernandez, Juan Emilio Losa García, Ana Cerezales Calviño, María Elisa Pino Díaz, Clara Martínez Montes, Víctor Arenas García, Francisco Arnaiz de las Revillas, Hadrián Pernas Pardavila, Sergio Padilla, María Ángeles Garcinuño Jiménez, Lucía Alonso Alonso, Noemí Ramos Vicente, Patricia Noemí Barragán Gallo, Rebeca Cabo Magadan, Míkel del Álamo, Miguel Vicente Egido Murciano, Alberto Juárez Toquero, Alberto Romero Palacios, Marta Clavero Olmos, María del Mar García Navarro, José Sanz, Juan Carlos Gainzarain, Marta Milian Sanz, Beatriz de la Calle, Oscar Luis Ferrero Benéitez, Jesús Troya, Luis Buzón‐Martín, The RELATIVITY Group

**Affiliations:** ^1^ Vega Baja Hospital Orihuela Spain; ^2^ Department of Clinical Medicine Miguel Hernández University Elche Spain; ^3^ CIBERINFEC Carlos III Health Institute Madrid Spain; ^4^ La Paz University Hospital IdiPaz Madrid Spain; ^5^ Fuenlabrada University Hospital Madrid Spain; ^6^ Fundación Para La Investigación e Innovación Biomédica (FIIB) Del Hospital Universitario Infanta Leonor y Del Hospital Universitario Del Sureste Madrid Spain; ^7^ Faculty of Health Sciences International University of La Rioja (UNIR) Logroño Spain; ^8^ I+12 Research Institute 12 De Octubre University Hospital Madrid Spain; ^9^ Infanta Leonor University Hospital Madrid Spain; ^10^ Guadalajara University Hospital Guadalajara Spain; ^11^ Castilla La Mancha Research Institute (IDISCAM) Alcalá University Alcalá de Henares Spain; ^12^ Valencia Clinical University Hospital Valencia Spain; ^13^ Son Llatzer University Hospital Palma de Mallorca Spain; ^14^ Son Espases University Hospital Palma de Mallorca Spain; ^15^ Lozano Blesa Clinical University Hospital Zaragoza Spain; ^16^ Puerta De Hierro University Hospital Madrid Spain; ^17^ Health Research Institute Fundación Jiménez Díaz (IIS‐FJD, UAM) Infectious Diseases Division, Fundación Jiménez Díaz University Hospital Madrid Spain; ^18^ Infectious Diseases Division Burgos Hospital Burgos Spain; ^19^ Denia Marina Salud Hospital Alicante Spain; ^20^ Parc Taulí University Hospital Sabadell Spain; ^21^ Infectious Diseases Division La Princesa Hospital Madrid Spain; ^22^ Bellvitge University Hospital Barcelona Spain; ^23^ Miguel Servet University Hospital Zaragoza Spain; ^24^ Department of Infectious Diseases University Hospital Ramón y Cajal, IRYCIS Madrid Spain; ^25^ Torrejón University Hospital Madrid Spain; ^26^ Reina Sofía General University Hospital Murcia Spain; ^27^ Internal Medicina-Infectious Disease Unit IdISS Clínico San Carlos Hospital Madrid Spain; ^28^ Universidad Complutense de Madrid Madrid Spain; ^29^ Álvaro Cunqueiro University Hospital Vigo Spain; ^30^ Mutua De Terrassa University Hospital Barcelona Spain; ^31^ Toledo University Hospital Toledo Spain; ^32^ Canarias University Hospital Tenerife Spain; ^33^ Morales Meseguer General University Hospital Murcia Spain; ^34^ Gregorio Marañón General University Hospital Madrid Spain; ^35^ Hospital Verge de La Cinta De Tortosa Tarragona Spain; ^36^ Segovia University Care Complex Segovia Spain; ^37^ Santa Caterina de Salt Hospital Girona Spain; ^38^ Virgen De Las Nieves University Hospital and Granada Biomedical Research Institute Granada Spain; ^39^ Hospital Central de La Defensa Gómez Ulla Madrid Spain; ^40^ Viladecans Hospital Barcelona Spain; ^41^ Alcorcón University Hospital Madrid Spain; ^42^ Rey Juan Carlos University Madrid Spain; ^43^ Doctor José Molina Orosa University Hospital Las Palmas Spain; ^44^ San Agustín University Hospital Asturias Spain; ^45^ San Cecilio Clinical Hospital Granada Spain; ^46^ Cabueñes University Hospital Asturias Spain; ^47^ Infectious Diseases Service Marqués De Valdecilla‑IDIVAL University Hospital Santander Spain; ^48^ Pontevedra University Hospital Complex Galicia Spain; ^49^ Infectious Diseases Unit Hospital General Universitario de Elche Alicante Spain; ^50^ Complejo Asistencial de Ávila Ávila Spain; ^51^ Hospital De Jove Asturias Spain; ^52^ Obispo Polanco Hospital Teruel Spain; ^53^ Hospital Residencia Sant Camil de Sant Pere De Ribes Barcelona Spain; ^54^ Central De Asturias University Hospital Oviedo Spain; ^55^ Cruces University Hospital Vizcaya Spain; ^56^ San Jorge University Hospital Huesca Spain; ^57^ Río Hortega de Valladolid Hospital Valladolid Spain; ^58^ Puerto Real University Hospital Cádiz Spain; ^59^ Infanta Elena University Hospital Madrid Spain; ^60^ Vinalopo University Hospital Alicante Spain; ^61^ Príncipe De Asturias University Hospital Madrid Spain; ^62^ Álava University Hospital Vitoria‐Gasteiz Spain; ^63^ Hospital De Valls Tarragona Spain; ^64^ Nuestra Señora del Prado General Hospital Talavera de la Reina Spain; ^65^ Basurto University Hospital Bilbao Spain

**Keywords:** antiretroviral treatment, cabotegravir, effectiveness, health access, HIV, long‐acting, migration, persistence

## Abstract

**Introduction:**

Migrants living with HIV often face high mobility, vulnerability and limited baseline information on HIV‐1 genotype or treatment history. We aimed to assess the effectiveness and persistence of long‐acting injectable cabotegravir and rilpivirine (LAI CAB+RPV) among migrants in Spain.

**Methods:**

This multicentre cohort study across 58 Spanish hospitals included virologically suppressed adults switching to CAB+RPV LAI before January 2025. Data collection started in June 2023. Baseline characteristics and outcomes were compared by migrant status, and multivariate Cox proportional hazards regression models were fitted to assess factors associated with virological failure (VF) and discontinuation. Propensity score matching (PSM) by gender, age, known genotype and prior VF was employed to control for confounding.

**Results:**

Of 3135 participants, 951 (30.3%) were migrants, predominantly from Latin America. Median follow‐up was 13.8 months (interquartile range 8.91–19.1). VF occurred in 0.9% of migrants versus 0.5% of Spanish‐born individuals (odds ratio 1.89, 95% confidence interval [CI] 0.69–5.03; *p* = 0.22). In adjusted models, migrant status showed a non‐significant trend towards higher VF (adjusted hazard ratio [aHR] 2.16, 95% CI 0.89–5.22; *p* = 0.079). At 12 months, 95.8% of migrants (461/481) persisted on LAI CAB+RPV treatment versus 98.3% of Spanish‐born individuals (1348/1372) (*p* = 0.005). Discontinuation due to any adverse event was more frequent in migrants (3.3% vs. 1.8%). Migrant status was significantly associated with discontinuation due to both local (aHR 2.63, 95% CI 1.33–5.26; *p* = 0.005) and systemic adverse events (aHR 3.33, 95% CI 1.45–7.69, *p* = 0.005).

In the PSM cohort (*n* = 932 per group), migrant status was independently associated with increased risk of VF (aHR 3.51, 95% CI 0.95–12.98, *p* = 0.045) and discontinuation due to systemic adverse events (aHR 2.88, 95% CI 1.01–8.17, *p* = 0.047).

**Conclusions:**

Nearly one‐third of participants switching to LAI CAB+RPV were migrants. While VF was rare overall, migrants had a significantly higher risk of treatment discontinuation, partly driven by adverse events. These findings highlight the need for closer monitoring and tailored strategies to optimize persistence with LAI regimens in migrant populations.

**Abbreviations**
ART: antiretroviral treatment
BMI: body mass index
CAB: cabotegravir
CI: confidence interval
DOR: doravirine
DRV/cob/FTC/TAF: darunavir/cobicistat/emtricitabine/tenofovir alafenamide
DTG/3TC: dolutegravir/lamivudine
DTG/RPV: dolutegravir/rilpivirine
GBMSM: gays, bisexuals and other men who have sex with men
INSTI: integrase strand transfer inhibitor
IQR: interquartile range
LAI: long‐acting injectable
NA: not available/not applicable
NNRTI: non‐nucleoside reverse transcriptase inhibitor
NRTI: nucleoside reverse transcriptase inhibitor
OR: odds ratio
PI: protease inhibitor
PWH: people living with HIV
RAL+FTC/TAF: raltegravir+emtricitabine/tenofovir alafenamide
RAM: resistance‐associated mutation
RPV: rilpivirine
VF: virological failure
VL: viral load
WT: wild type

## 1 Introduction

Migrants are a key population living with HIV globally and across Europe. In 2023, 11,837 people born outside their reporting country were diagnosed with HIV in the European Economic Area, accounting for 47.9% of new cases [1]. Main areas of origin include sub‐Saharan Africa, Central and Eastern Europe, and Latin America. Migrants living with HIV experience intersecting stigmas related to HIV, migrant status, race and gender [2]. Despite a lack of granular information on the continuum of care for these populations [3], migrants frequently face job insecurity, healthcare barriers, and high mobility within and outside the host country. Consequently, antiretroviral therapy (ART) coverage and suppression rates are often lower in vulnerable groups, particularly undocumented migrants [4]. Evidence shows that migrants—especially from sub‐Saharan Africa—are more likely to experience ART discontinuation, loss to follow‐up, and virological and immunological failure compared to the general population [5].

In Spain, 49.8% of the 3196 people diagnosed with HIV in 2023 were born abroad, two‐thirds of whom originated from Latin America [6]. This population, including individuals from Venezuela, Colombia or Peru, encounters significant barriers to care, as evidenced by the high proportion of treatment interruptions (21%) and loss of viral suppression (33%) among asylum seekers [7].

Cabotegravir plus rilpivirine (CAB+RPV) is the first long‐acting injectable (LAI) ART approved in Europe. Administered intramuscularly every 8 weeks, it has demonstrated high efficacy in clinical trials as a switching strategy for virologically suppressed people living with HIV (PWH) [8, 9]. Real‐world cohort studies have confirmed its safety, efficacy and acceptability [10, 11, 12, 13]. Notably, LAI regimens can overcome adherence hurdles, HIV stigma and treatment fatigue associated with lifelong daily oral therapy [14, 15].

Risk factors for virological breakthrough include a body mass index (BMI) ≥30 kg/m^2^, non‐nucleoside reverse transcriptase inhibitor (NNRTI) resistance‐associated mutations (RAMs) and HIV‐1 A1/A6 subtypes [16], though the CARES study showed low virological failure (VF) rates in the A1 variant (0.8%) [17]. Migrants may face higher risks of failure due to a higher prevalence of non‐B subtypes, high mobility and vulnerability, and incomplete previous genotype and ART information. However, LAI regimens may offer advantages regarding work‐schedule compatibility and reduced stigmatization.

Migrants represent a population where clinical and social dimensions converge. Factors such as geographic mobility, fragmented treatment histories, and underrepresentation in clinical trials highlight the need to evaluate innovative regimens such as LAI CAB+RPV through a health equity lens. Ensuring therapeutic benefits are distributed fairly is essential.

This study aimed to evaluate the effectiveness and persistence of CAB+RPV LAI among migrants in Spain compared with Spanish‐born individuals, identifying whether migrant status is associated with higher rates of VF or discontinuation to inform tailored interventions.

## 2 Methods

### 2.1 Study Design and Setting

The RELATIVITY study is a nationwide, multicentre, prospective cohort study designed to evaluate the effectiveness and safety of LAI CAB+RPV in 58 real‐world clinical settings in Spain. The study was launched in June 2023, although LAI CAB+RPV was commercialized in Spain in December 2022, and monitoring is expected to continue until 2029. Patients switching to LAI CAB+RPV before January 2025 were included. Patient information, until the time the participant's hospital was activated in the cohort and the patient signed informed consent, was collected retrospectively; from that point onwards, it was collected prospectively. The data have been reported elsewhere [18]; we present here a substudy of the RELATIVITY cohort aiming to evaluate effectiveness and treatment persistence in migrants and to compare it to those of Spanish‐born individuals living with HIV. We adopted the pragmatic definition of migrant as foreign‐born, consistent with national HIV surveillance reports.

### 2.2 Study Participants

Virologically suppressed adults (≥18 years) living with HIV who had received at least one dose of LAI CAB+RPV before January 2025 and who had an undetectable viral load (<50 copies/mL) at the time of treatment initiation were invited to participate. Exclusion criteria included pregnancy and prior participation in LAI CAB+RPV clinical trials. Pregnant women were excluded because LAI was not recommended during pregnancy in the product label or in the current European/Spanish guidelines. Participants were divided into two main study groups according to country of birth (migrants vs. Spanish‐born).

For the primary analysis, all participants included in the cohort had an undetectable viral load (<50 copies/mL) at the start of LAI CAB+RPV. Additionally, a secondary analysis of outcomes was conducted considering only those participants who met the approved label criteria for initiating LAI CAB+RPV (on‐label group), which included an undetectable viral load, the absence of RAMs to integrase strand transfer inhibitors (INSTIs) and NNRTIs, and no prior VF on these drug classes.

### 2.3 Data Collection

Study data were collected and managed using REDCap (Research Electronic Data Capture), hosted at Infanta Leonor University Hospital [19]. Information collected included demographic data, anthropometric measurements, HIV‐related information, laboratory data, treatment information (injection schedule, type and site of injection and oral bridge therapies) and follow‐up. There was no established follow‐up schedule; each hospital followed its own standard procedures for assessments, in accordance with national and international guidelines.

### 2.4 Variables

Data collected included patient demographics, comorbidities, substance use, weight and BMI. Information on HIV diagnosis, previous ART regimens, VF and previous blips was collected, along with HIV‐1 subtype and genotype resistance testing when available. A viral blip was defined as a transient, low‐level increase in HIV‐1 RNA (50–200 copies/mL) that returned to undetectable levels without therapy modification. VF was defined as two consecutive HIV‐1 RNA measurements of ≥200 copies/mL or a single measurement of >500 copies/mL leading to treatment discontinuation. The primary outcome was 12‐month persistence. Secondary outcomes included virological suppression (<50 copies/mL), VF, immunological markers, adherence to the injection schedule and reasons for discontinuation. Adherence was assessed as the proportion of days covered. Doses were considered on time if administered within the ±7 days window of the 8‐week injection schedule. Treatment persistence was defined as the time from treatment initiation to permanent discontinuation of the ART regimen. All variables and outcomes were measured in the real‐world clinical context of each participating centre.

### 2.5 Statistical Analysis

Categorical variables are presented as absolute frequencies and percentages, and quantitative variables as median (interquartile range, IQR), as the normality test (Kolmogorov−Smirnov or Shapiro−Wilk test) showed a non‐parametric distribution in all cases. Comparative analyses were undertaken using the Chi‐square test or Fisher's exact test for categorical variables, and the independent samples *t*‐test or the Mann−Whitney U test for quantitative variables, as appropriate. Kaplan−Meier survival curves were generated to estimate the probability of remaining free from VF, side effects or other discontinuations over time, with analyses comparing migrants and Spanish‐born participants using the log‐rank test. To quantify associations between covariates and time‐to‐event outcomes, we fitted multivariable Cox proportional hazards regression models and report results as adjusted hazard ratios (aHR) with 95% confidence intervals (CI) and *p* values. All Cox proportional hazards models included a comprehensive set of potential predictors, comprising baseline age and gender, nationality, prior VF, the presence of previous antiretroviral resistance and a wide range of baseline comorbidities. The proportional hazards assumption was assessed by visual inspection of Schoenfeld residuals and formal testing, showing no evidence of violation. Model performance was evaluated by the concordance index, and overall significance was confirmed by the likelihood ratio test. Propensity score matching (PSM) was employed to control for confounding, matching individuals by gender, age, known genotype and prior VF. Standardized mean differences before and after matching were calculated to assess covariate balance, considering values <0.1 as indicative of good balance. Missing data were not imputed. The registry underlying this cohort is highly exhaustive, and missingness corresponds exclusively to clinical information unavailable in the patients’ medical records. We performed a secondary analysis including only PWH in whom the prescription was in accordance with the technical sheet, excluding those who had NNRTI or INSTI mutations (the on‐label cohort). All analyses were conducted using R version 4.4.0 (R Foundation for Statistical Computing, Vienna, Austria).

### 2.6 Ethical Statement

The study protocol was approved by the Ethics Committee for Research with Medicines of the Burgos and Soria Health Area (code 23–00144). All patients received an information sheet and provided written informed consent before enrolment. Data confidentiality was maintained in compliance with Spanish and European Union data protection regulations.

## 3 Results

### 3.1 Study Population

**FIGURE 1:**
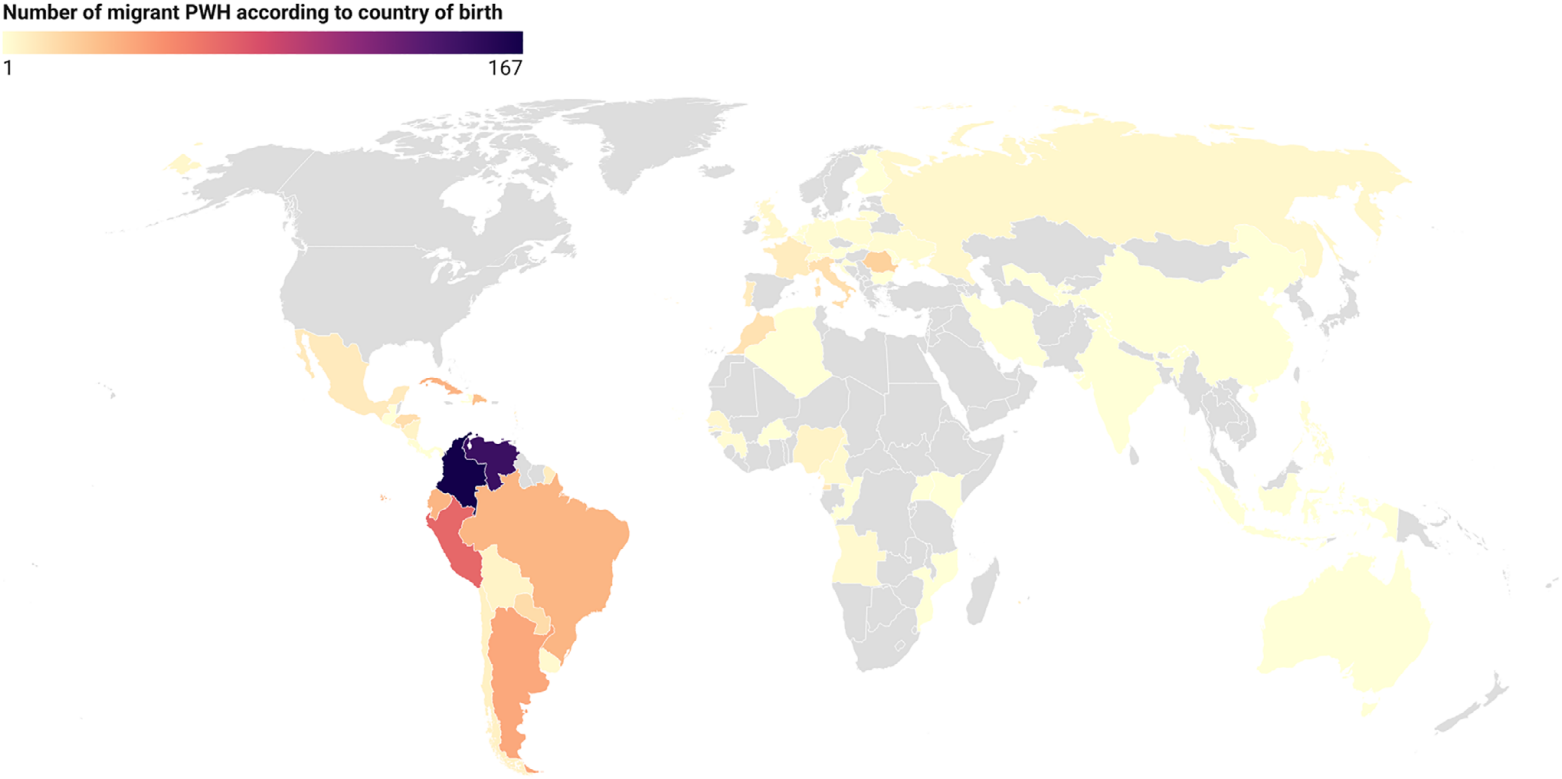
Country of origin in migrant participants switching to cabotegravir plus rilpivirine long‐acting injectable in the RELATIVITY cohort, Spain. PWH: people living with HIV.

Of the 3192 individuals registered in the RELATIVITY cohort who switched to LAI CAB+RPV before January 2025, 3135 met selection criteria for inclusion in the study. Of those, 951 (30.3%) were migrants, mainly from Latin America (*n* = 766, 80.5%), Africa (*n* = 63, 6.6%) and Western Europe (*n* = 51, 5.4%). Detailed geographic origins are shown in Figure 1 and File S1 (Table S1).

Baseline characteristics differed between migrant and Spanish‐born individuals and are detailed in Table 1. Compared to those born in Spain, migrants living with HIV had a lower median age (39.8 vs. 47.6 years; *p*<0.001), a higher proportion of cisgender women (18.2% vs. 13.3%; *p* = 0.001), a shorter median duration of years on ART prior to switching to LAI (7 vs. 10 years; *p*<0.001) and less frequently documented prior VF (2.1% vs. 4.1%; *p* = 0.004). Reasons for switching did not differ between groups.

**TABLE 1:** Baseline characteristics in migrant and Spanish‐born people living with HIV and switching to long‐acting injectable cabotegravir plus rilpivirine in the RELATIVITY cohort, Spain.

Variables	Migrants (*N* = 951)	Spanish‐born (*N* = 2184)	OR (95% CI)	*p*‐value
Age in years, median (IQR)	39.8 (33.0, 48.0)	47.6 (40.0, 56.0)	NA	**<0.001**
Gender				
Cisgender man	763/947 (80.6%)	1882/2178 (86.4%)	0.65 (0.53−0.8)	**<0.001**
Cisgender woman	172/947 (18.2%)	290/2178 (13.3%)	1.44 (1.17−1.78)	**0.001**
Transgender woman	12/947 (1.3%)	6/2178 (0.3%)	4.65 (1.61−15.13)	**0.002**
Body mass index, kg/m^2^, median (IQR)	25.1 (22.0, 27.8)	24.6 (21.8, 27.5)	NA	0.097
Viral load (copies/mL) at HIV diagnosis, median (IQR)	39,816.0 (6597.8, 141,864.2)	55,700.0 (123,36.0, 205,461.0)	NA	**<0.001**
Months from diagnosis to first ART initiation, median (IQR)	1.0 (0.2, 9.0)	2.0 (0.5, 21.7)	NA	**<0.001**
Years on ART from treatment initiation to start of CAB+RPV, median (IQR)	7.0 (4.0, 12.0)	10.0 (6.0, 16.0)	NA	**<0.001**
CD4 nadir, cells/mm^3^, median (IQR)	325.0 (199.0, 495.8)	346.0 (192.0, 500.0)	NA	0.639
Baseline CD4, cells/mm^3^, median (IQR)	741.0 (547.5, 935.5)	805.0 (616.0, 1042.5)	NA	**<0.001**
Drug use				
Any drug use	237/867 (27.3%)	717/2061 (34.8%)	0.71 (0.59−0.84)	**<0.001**
Smoking	184/951 (19.3%)	744/2184 (34.1%)	0.46 (0.38−0.56)	**<0.001**
Alcohol	104/951 (10.9%)	203/2184 (9.3%)	1.2 (0.92−1.55)	0.170
Chemsex	40/951 (4.2%)	74/2184 (3.4%)	1.25 (0.82−1.88)	0.256
Other recreational drug use	54/951 (5.7%)	111/2184 (5.1%)	1.12 (0.79−1.59)	0.487
HIV transmission route				
Unprotected sex (GBMSM)	635/951 (66.8%)	1330/2184 (60.9%)	1.29 (1.1−1.52)	**0.002**
Unprotected sex (heterosexual)	192/951 (20.2%)	396/2184 (18.1%)	1.14 (0.94−1.39)	0.179
Intravenous drug use	9/951 (0.9%)	186/2184 (8.5%)	0.1 (0.05−0.2)	**<0.001**
Vertical	6/951 (0.6%)	21/2184 (1%)	0.65 (0.22−1.68)	0.408
Other	12/951 (1.3%)	28/2184 (1.3%)	0.98 (0.45−2.01)	1.000
NA	97/951 (10.2%)	223/2184 (10.2%)	1 (0.77−1.29)	1.000
Prior AIDS diagnosis				
Yes	96/951 (10.1%)	293/2184 (13.4%)	0.72 (0.56−0.93)	**0.009**
No	792/951 (83.3%)	1765/2184 (80.8%)	1.18 (0.96−1.46)	0.109
NA	63/951 (6.6%)	126/2184 (5.8%)	1.16 (0.83−1.6)	0.369
Previous VF to any ART regimen				
Yes	20/951 (2.1%)	90/2184 (4.1%)	0.5 (0.29−0.82)	**0.004**
No	768/951 (80.8%)	1818/2184 (83.2%)	0.84 (0.69−1.03)	0.102
NA	163/951 (17.1%)	276/2184 (12.6%)	1.43 (1.15−1.77)	**0.001**
Third drug at the time of VF				
INSTI	8/19 (42.1%)	18/72 (25%)	2.18 (0.65−7.03)	0.161
NNRTI	4/19 (21.1%)	22/72 (30.6%)	0.61 (0.13−2.22)	0.571
PI	7/19 (36.8%)	32/72 (44.4%)	0.73 (0.22−2.3)	0.611
Number of blips in the 5 years prior to CAB/RPV treatment				
0	670/833 (80.4%)	1659/2003 (82.8%)	0.85 (0.69−1.06)	0.132
1	97/833 (11.6%)	217/2003 (10.8%)	1.08 (0.83−1.41)	0.554
2	31/833 (3.7%)	62/2003 (3.1%)	1.21 (0.75−1.91)	0.418
3	14/833 (1.7%)	27/2003 (1.3%)	1.25 (0.6−2.49)	0.493
≥4	21/833 (2.5%)	38/2003 (1.9%)	1.34 (0.74−2.35)	0.312
Detailed history of ART adherence and VFs (yes)	373/454 (82.2%)	788/879 (89.6%)	0.53 (0.38−0.75)	**<0.001**
Third agent of ART prior to inclusion				
INSTI	763/951 (80.2%)	1656/2184 (75.8%)	1.29 (1.07−1.57)	**0.007**
NNRTI	89/951 (9.4%)	238/2184 (10.9%)	0.84 (0.65−1.1)	0.204
PI	40/951 (4.2%)	131/2184 (6%)	0.69 (0.47−1)	**0.049**
Others	18/951 (1.9%)	68/2184 (3.1%)	0.6 (0.33−1.03)	0.057
NA	41/951 (4.3%)	91/2184 (4.2%)	1.04 (0.69−1.53)	0.847
Reason for switching				
Toxicity	11/951 (1.2%)	37/2184 (1.7%)	0.68 (0.31−1.37)	0.342
Drug interaction	4/951 (0.4%)	8/2184 (0.4%)	1.15 (0.25−4.3)	0.762
Simplification	188/951 (19.8%)	555/2184 (25.4%)	0.72 (0.6−0.87)	**0.001**
Comfort/quality of life	483/951 (50.8%)	1123/2184 (51.4%)	0.98 (0.83−1.14)	0.756
Malabsorption	9/951 (0.9%)	33/2184 (1.5%)	0.62 (0.26−1.34)	0.239
Swallowing disorders	3/951 (0.3%)	13/2184 (0.6%)	0.53 (0.1−1.93)	0.419
Patient request	362/951 (38.1%)	790/2184 (36.2%)	1.08 (0.92−1.27)	0.314

Abbreviations: ART, antiretroviral treatment; CAB, cabotegravir; CI, confidence interval; GBMSM, gay, bisexual and other men who have sex with men; INSTI, integrase strand transfer inhibitor; IQR, interquartile range; LAI, long‐acting injectable; NA, not available; NNRTI, non‐nucleoside reverse transcriptase inhibitor; NRTI, nucleoside reverse transcriptase inhibitor; OR, odds ratio; PI, protease inhibitor; RPV, rilpivirine; VF, virological failure.

**In bold**: statistically significant (*p*<0.05).

Data on resistance testing prior to the switch were less frequently available in migrants (40.2% vs. 49.4%; *p*<0.001). Among the sample with available data, most participants had no detectable resistance mutations, with similar proportions between groups (84.3% in migrants vs. 85.6% in Spanish‐born; *p* = 0.56). The prevalence of mutations affecting INSTIs was also similar: 2.1% in migrants versus 0.9% in Spanish‐born individuals (*p* = 0.101). NNRTI resistance mutations were identified in 9.9% versus 8.2% (*p* = 0.34), and only the E138A mutation was statistically more frequent among migrants. Further details on genotype resistance patterns, including specific mutations and HIV‐1 subtypes, are provided in Table 2.

**TABLE 2:** Genotype resistance patterns, specific mutations and HIV‐1 subtypes in migrant and Spanish‐born people living with HIV and switching to long‐acting injectable cabotegravir plus rilpivirine in the RELATIVITY cohort, Spain.

	Migrants (*N* = 951)	Spanish‐born (*N* = 2184)	OR (95% CI)	*p*‐value
Genotype available	382 (40.2%)	1079 (49.4%)	0.69 (0.59−0.8)	**<0.001**
Subtype				
B	182/382 (47.6%)	482/1079 (44.7%)	1.13 (0.89−1.43)	0.339
A1/A2	3/382 (0.8%)	25/1079 (2.3%)	0.33 (0.06−1.1)	0.080
F/CRF	19/382 (5%)	26/1079 (2.4%)	2.12 (1.09−4.03)	**0.016**
Other	14/382 (3.7%)	45/1079 (4.2%)	0.87 (0.44−1.64)	0.763
Not available	164/382 (42.9%)	501/1079 (46.4%)	0.87 (0.68−1.11)	0.256
Wild type (no mutations)	255/382 (66.8%)	754/1079 (69.9%)	0.87 (0.67−1.12)	0.274
NRTI resistance mutations	27/382 (7.1%)	102/1079 (9.5%)	0.73 (0.45−1.15)	0.173
*184 V*	7/382 (1.8%)	22/1079 (2%)	0.9 (0.32−2.2)	1.000
*Other nucleoside analogue mutations*	21/382 (5.5%)	78/1079 (7.2%)	0.75 (0.43−1.24)	0.287
NNRTI resistance mutations	38/382 (9.9%)	89/1079 (8.2%)	1.23 (0.8−1.86)	0.341
*K103N*	13/382 (3.4%)	30/1079 (2.8%)	1.23 (0.58−2.46)	0.597
*E138A*	5/382 (1.3%)	3/1079 (0.3%)	4.76 (0.92−30.74)	**0.033**
*Other NNRTI mutations*	22/382 (5.8%)	58/1079 (5.4%)	1.08 (0.62−1.82)	0.794
Integrase resistance mutations	8/382 (2.1%)	10/1079 (0.9%)	2.29 (0.78−6.49)	0.101
*L74M/I/F*	1/382 (0.3%)	0/1079 (0%)	8.49 (0.53−Inf)	0.069
*T97A*	0/382 (0%)	1/1079 (0.1%)	0.94 (0−15.06)	1.000
*Other integrase mutations*	7/382 (1.8%)	9/1079 (0.8%)	2.22 (0.7−6.75)	0.148
Total number of detected mutations				
0	322/382 (84.3%)	924/1079 (85.6%)	0.9 (0.65−1.27)	0.556
1	50/382 (13.1%)	116/1079 (10.8%)	1.25 (0.86−1.8)	0.223
2	9/382 (2.4%)	38/1079 (3.5%)	0.66 (0.28−1.41)	0.314
3	1/382 (0.3%)	1/1079 (0.1%)	2.83 (0.04−221.9)	0.455

Abbreviations: CI, confidence interval; NNRTI, non‐nucleoside reverse transcriptase inhibitor; NRTI, nucleoside reverse transcriptase inhibitor; OR, odds ratio.

**In bold**: statistically significant (*p*<0.05).

### 3.2 Outcomes

Treatment adherence, virological outcomes and treatment discontinuation rates for both groups are shown in Table 3.

**TABLE 3:** Persistence, adherence, discontinuation and adverse effects for migrant and Spanish‐born individuals switching to cabotegravir plus rilpivirine long‐acting injectable in the RELATIVITY cohort, Spain.

	Migrants (*N* = 951)	Spanish‐born (*N* = 2184)	OR (95% CI)	*p*‐value
Months of follow‐up, median (IQR)	12.4 (7.0, 18.2)	14.9 (9.1, 19.1)	NA	**<0.001**
Therapeutic adherence, percentage of days covered				
<90%	13/919 (1.4%)	37/2106 (1.8%)	0.8 (0.39−1.55)	0.540
90−99.9%	137/919 (14.9%)	288/2106 (13.6%)	1.1 (0.89−1.38)	0.897
100%	769/919 (83.7%)	1781/2106 (84.6%)	0.94 (0.75−1.16)	0.550
Viral load at follow‐up				
<50 copies/mL at month 7	377/388 (97.2%)	864/882 (98%)	0.71 (0.32−1.69)	0.416
<50 copies/mL at month 13	200/210 (95.2%)	601/616 (97.6%)	0.5 (0.21−1.26)	0.103
Treatment discontinuation	102/951 (10.7%)	127/2184 (5.8%)	1.95 (1.48–2.56)	**<0.001**
Temporary	17/951 (1.8%)	14/2184 (0.6%)	2.82 (1.3−6.21)	**0.005**
Definitive	85/951 (8.9%)	113/2184 (5.2%)	1.8 (1.33−2.43)	**<0.001**
LAI CAB+RPV persistence at 6 months	706/757 (93.3%)	1844/1919 (96.1%)	0.56 (0.38–0.84)	**0.003**
LAI CAB+RPV persistence at 12 months	461/481 (95.8%)	1348/1372 (98.3%)	0.41 (0.2–0.85)	**0.005**
Months to definitive treatment discontinuation, median (IQR)	7.0 (3.2, 11.3)	8.7 (4.3, 11.4)	NA	0.307
Reasons for permanent treatment discontinuation				
VF	9/951 (0.9%)	11/2184 (0.5%)	1.89 (0.69−5.03)	0.220
Any adverse event	32/951 (3.4%)	41/2184 (1.8%)	1.88 (1.21–2.92)	**0.003**
*Local injection‐site reaction*	19/951 (2%)	25/2184 (1.1%)	1.76 (0.91−3.35)	0.070
*Systemic adverse effects*	13/951 (1.4%)	16/2184 (0.7%)	1.88 (0.83−4.18)	0.104
Other reasons for discontinuation	48/951 (5%)	65/2184 (3%)	1.73 (1.16−2.58)	**0.006**
*Change of residence or transfer*	21/951 (2.2%)	17/2184 (0.8%)	2.88 (1.51–5.48)	**0.001**
*Logistical issues or poor adherence*	11/951 (1.2%)	11/2184 (0.5%)	2.31 (1.00–5.35)	**0.044**
*Pregnancy or pregnancy intention*	4/951 (0.4%)	2/2184 (0.1%)	4.61 (0.84–25.2)	0.053
*Medical decision, patient decision or unspecified issues*	10/951 (1.1%)	30/2184 (1.4%)	0.76 (0.37–1.57)	0.460

Abbreviations: CI, confidence interval; NA, not applicable; OR, odds ratio; VF, virological failure; VL, viral load.

**In bold**: statistically significant (*p*<0.05).

#### 3.2.1 Virological Outcomes

**FIGURE 2:**
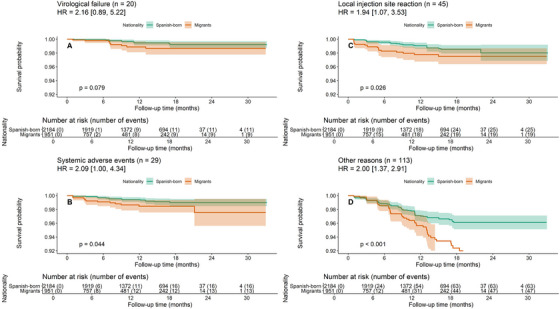
(A) Virological failure, (B) systemic adverse events, (C) local injection site reactions, or (D) other reason for discontinuations in migrants and Spanish‐born people living with HIV starting cabotegravir plus rilpivirine long‐acting injectable in the RELATIVITY cohort. HRs displayed in the figure are for migrants.

Rates of VF were low in both groups, with no statistically significant difference observed between migrants and Spanish‐born individuals. VF occurred in 0.9% of migrants compared to 0.5% of Spanish‐born individuals (odds ratio [OR] 1.89, 95% CI 0.69–5.03; *p* = 0.22). Kaplan−Meier survival analysis (Figure 2) showed no significant difference in the probability of VF over time between groups (log‐rank *p* = 0.86).

In the multivariate Cox proportional hazards regression model, migrant status showed a significant trend towards higher VF (aHR 2.16; 95% CI: 0.89–5.22; *p* = 0.079), limited by the low number of events and imprecise estimates (File S2, Table S2).

#### 3.2.2 Factors Associated With VF Among Migrants

Table 4 shows the characteristics of patients in the cohort experiencing VF, including emerging RAMs. Among migrants, VF was associated with a higher baseline BMI and a higher HIV viral load at diagnosis. A history of at least three blips in the 5 years prior to switching to LAI CAB+RPV was more frequent among those experiencing VF (Table 5).

**TABLE 4:** Characteristics of patients experiencing VF after switching to long‐acting injectable cabotegravir plus rilpivirine in the RELATIVITY cohort, Spain.

Country of origin	Gender, age	HIV transmission route	CD4 nadir, cells/mm^3^	VL at diagnosis, copies/mL	Previous AIDS diagnosis	Months dx‐ ART initiation	Years on ART when starting CAB+RPV	Months with undetectable VL before switching to LAI CAB+RPV	Previous VF	*N* blips in the 5 years prior to CAB+RPV	ART prior to switch	Baseline BMI, kg/m^2^	Baseline CD4, cells/mm^3^	Baseline CD4/CD8 ratio	Previous RAMs	New RAMs	Oral ART after VF	Undetectable VL after oral ART
Spain	M, 57	Htx	123	500,000	No	NA	16	179	Yes	0	RAL+FTC/TAF	NA	629	1.24	NRTI: 184V	INSTI: 63P	DTG/3TC+DOR	No
Spain	M, 35	GBMSM	NA	NA	No	1	15	192	No	0	DTG/RPV	27.75	770	0.97	—	INSTI: E138K, N155H	DRV/cob/ABC/3TC	Yes
Spain	M, 46	NA	233	NA	No	33	10	109	No	0	BIC/FTC/TAF	23.36	1488	0.92	NRTI: V90I	INSTI: Q148R	BIC/FTC/TDF	Yes
Spain	F, 52	Htx	98	NA	No	36	23	108	Yes	0	DTG/RPV	36.37	460	0.50	NRTI: 184 V; NNRTI: K103N	—	DRV/cob/FTC/TAF	Yes
Spain	M, 55	GBMSM	573	68,800	No	22	10	115	No	0	BIC/FTC/TAF	29.07	1162	1.57	WT	NNRTI: E138A, V179E, P225H, F227L, N348I	DRV/cob/FTC/TAF	No
Spain	M, 43	GBMSM	160	16,500	No	1	11	10	No	1	BIC/FTC/TAF	23.44	778	1.04	WT	NNRTI: K101E; INSTI: Q146L	BIC/FTC/TAF	No
Spain	M, 46	GBMSM	1382	44.6	No	0	2	24	No	0	BIC/FTC/TAF	24.57	1432	2.55	—	—	BIC/FTC/TAF	Yes
Spain	M, 40	GBMSM	669	296	No	66	7	84	No	0	DTG/3TC	25.76	1064	1.09	WT	—	DRV/cob/FTC/TAF	Yes
Spain	F, 59	NA	5	NA	NA	0	21		NA	> 3	BIC/FTC/TAF	NA			—	INSTI: G140S, L74M, Q148H	DRV/cob/FTC/TAF	Yes
Spain	M, 40	GBMSM	NA	NA	No	56	4	3	No	3	DTG/ABC/3TC	24.32	1193	0.98	WT	—	BIC/FTC/TAF	Yes
Spain	M, 62	GBMSM	275	108,000	No	1	8	84	No	0	DTG/3TC	25.11	704	0.38	—	NNRTI: K103N, E138K, N348I; INSTI: E138K, G140A, Q148R	DRV/cob/FTC/TAF	No
Equatorial Guinea	F, 23	Vertical	277	159,779	No	NA	12		No	0	BIC/FTC/TAF	28.65	514	1.58	—	—	DRV/cob/FTC/TAF	No
Colombia	M, 50	GBMSM	NA	NA	Yes	0	26	120	No	0	DTG/3TC	33.88	592	1.16	WT	NNRTI: L100I, K103N, L74M, T97A, G140S, Q148K, Q148R, E157Q	DRV/cob/FTC/TAF	Yes
Peru	M, 49	GBMSM	240	NA	No	24	7	7	No	3	BIC/FTC/TAF	22.5	510	0.69	WT	No	DRV/cob/FTC/TAF	Yes
Angola	M, 45	GBMSM	NA	NA	Yes	2	5	7	No	3	DTG/3TC	29.26	890	1.75	—	NNRTI: K103N, Y188L; INSTI: E138eK, Q148R, L74LM	DRV/cob/FTC/TAF	No
Peru	M, 28	GBMSM	146	132,589	No	1	1	7	No	0	BIC/FTC/TAF	28.17	625	0.54	—	—	DRV/cob/FTC/TAF	Yes
Paraguay	F, 41	Htx	42	128,096	Yes	3	5.7	66	No	0	DTG/RPV	39.65	892	1.08	WT	—	DRV/cob/FTC/TAF	Yes
Venezuela	M, 29	GBMSM	NA	NA	No	27	2	5	No	2	BIC/FTC/TAF	28.43	456	1.15	—	NNRTI: 74 M, 138K, 148KR	DRV/cob/FTC/TAF	No
Dominican Republic	F, 47	Htx	65	386,000	No	0	4	36	No	0	BIC/FTC/TAF	30.64	573	0.80	WT	NNRTI: K101Ke, V106Vi, Y181C)	BIC/FTC/TAF	No
Portugal	M, 48	Htx	425	97,500	No	0	6.2	69	No	0	DTG/3TC	26.65	833	0.90	WT	INSTI: E138K, Q148K	DRV/cob/FTC/TAF	Yes

Abbreviations: ART, antiretroviral treatment; BIC/FTC/TAF, bictegravir/emtricitabine/tenofovir alafenamide; CAB, cabotegravir; DOR, doravirina; DRV/cob/FTC/TAF, darunavir/cobicistat/emtricitabine/tenofovir alafenamide; DTG/ABC/3TC, dolutegravir/abacavir/lamivudine; DTG/3TC, dolutegravir/lamivudine; DTG/RPV, dolutegravir/rilpivirine; F, female; GBMSM, unprotected sex in gay, bisexual and other men who have sex with men; Htx, unprotected sex (heterosexual); INSTI, integrase strand transfer inhibitor; LAI, long‐acting injectable; M, male, NA, not available; NNRTI, non‐nucleoside reverse transcriptase inhibitor; RAL+FTC/TAF, raltegravir+ emtricitabine/tenofovir alafenamide; RPV, rilpivirine; VF, virological failure; VL, viral load; WT, wild type.

**TABLE 5:** Factors associated with VF in migrant individuals switching to long‐acting injectable cabotegravir plus rilpivirine in the RELATIVITY cohort, Spain.

	VF (*N* = 9)	No VF (*N* = 942)	OR (95% CI)	*p*‐value
Age, years, median (IQR)	45.0 (29.0, 48.0)	39.6 (33.0, 48.0)	NA	0.995
Cisgender women, *n* (%)	3/9 (33.3%)	169/938 (18.0%)	2.28 (0.36−10.76)	0.214
Sub‐Saharan African origin, *n* (%)	2/9 (22.2%)	60/930 (6.5%)	4.14 (0.41−22.34)	0.114
Baseline BMI, kg/m^2^, median, (IQR)	28.3 (27.7–30.4)	25.5 (23.5–28.1)	NA	**0.028**
CD4 nadir, cells/µL, median (IQR)	193.0 (85.2–267.8)	326.5 (199.0–498.5)	NA	0.065
VL at HIV diagnosis, copies/mL, median (IQR)	132,589 (128,096–159,779)	38,453 (6461–141,190)	NA	**0.044**
Prior AIDS diagnosis, *n* (%)	3/9 (33.3%)	93/942 (9.9%)	4.56 (0.72–21.74)	0.053
≥ 3 blips in the 5 years prior to switching to CAB+RPV LAI, *n* (%)	2/9 (22.2%)	12/824 (1.5%)	19.33 (1.76–115.78)	**0.009**
BIC/FTC/TAF regimen at baseline, *n* (%)	5/9 (55.6%)	254/942 (27.0%)	3.39 (0.90–12.71)	0.068

Abbreviations: BIC/FTC/TAF, bictegravir/emtricitabine/tenofovir alafenamide; BMI, body mass index; CAB, cabotegravir; IQR, interquartile range; LAI, long‐acting injectable; NA, not applicable; OR, odds ratio; RPV, rilpivirine; VF, virological failure; VL, viral load.

**In bold**: statistically significant (*p*<0.05).

#### 3.2.3 Treatment Persistence and Adherence

Median follow‐up for the cohort was 13.8 months (IQR 8.91−19.1). Overall adherence to LAI CAB+RPV was high, with no statistically significant difference in the proportion of individuals with less than 90% of days covered between groups (1.4% vs. 1.8%; *p* = 0.54). However, persistence rates at both 6 and 12 months were lower among migrants. At 6 months, 93.3% of migrants (706/757) persisted on LAI CAB+RPV treatment compared to 96.1% of Spanish‐born individuals (1844/1919) (*p* = 0.003), while at 12 months, persistence was 95.8% (461/481) among migrants and 98.3% among Spanish‐born individuals (1348/1372) (*p* = 0.005). Temporary LAI CAB+RPV interruptions were also more frequent in migrants (1.8% vs. 0.6%; *p* = 0.005).

Among those discontinuing LAI CAB+RPV, adverse events were the most common cause. Discontinuation due to any adverse event occurred more frequently in migrants (3.4% vs. 1.8%; *p* = 0.003). Other causes for discontinuation are shown in Table 3.

Kaplan−Meier estimates of time to key events, disaggregated by reason for treatment discontinuation, are shown in Figure 2. A multivariable Cox proportional hazards model was fitted to evaluate predictors of treatment discontinuation due to adverse events (File S2, Table S2). Older age (+1 year: aHR 1.05, 95% CI 1.01–1.09, *p* = 0.015), women (aHR 2.63, 95% CI 1.16–5.88, *p* = 0.021) and migrant status (aHR 3.33, 95% CI 1.45–7.69, *p* = 0.005) were associated with discontinuation due to systemic side effects. Local reactions were significantly associated with migrant status (aHR 2.63, 95% CI 1.33–5.26; *p* = 0.005) and hypertension (aHR 2.48; 95% CI 1.07–5.71; *p* = 0.034).

### 3.3 PSM Analysis

To control for potential confounding, a 1:1 propensity score‐matched cohort was generated based on gender, age, availability of baseline genotype and history of VF, yielding 932 individuals in each group (File S3, Tables S3.1−S3.4). In this matched cohort, VF remained infrequent and did not differ significantly between migrants and Spanish‐born individuals in the crude analysis (1% vs. 0.3%; OR 3.02, 95% CI 0.75–17.38; *p* = 0.15). However, definitive treatment discontinuation was more common among migrants (9% vs. 5.1%; OR 1.85; 95% CI 1.26–2.72; *p* = 0.001). Kaplan−Meier curves for time to VF and treatment discontinuation in the PSM cohort, stratified by specific reasons, are shown in File S3, Figure S3. In this case, the cumulative incidence of discontinuation was higher in migrants across all categories, including a borderline significant trend towards higher VF (aHR 3.51, 95% CI 0.95–12.98, *p* = 0.045), but with substantial uncertainty given the small number of failures.

### 3.4 On‐Label Analysis

The results of the secondary analysis, including only PWH in whom the prescription was in accordance with the technical sheet, and excluding those who had NNRTI or INSTI mutations, are presented in File S4, Tables S4.1−S4.4 and Figure S4.

## 4 Discussion

In our study, migrant PWH switching to LAI CAB+RPV exhibited low VF rates, comparable to those observed in pivotal clinical trials. However, they consistently showed higher discontinuation rates, partly due to local and systemic side effects, compared to the Spanish‐born population. These findings suggest that treatment persistence is a multidimensional outcome determined not only by pharmacological efficacy but also by clinical, organizational and patient‐experience factors.

Regarding VF, our analysis, using a definition aligned with recent consensus [20], consistently showed that the VF rate in migrants was ≤1%, similar to reports from clinical trials and other real‐world cohorts [12, 13, 21]. Crucially, in the context of sustained virological suppression, the absence of baseline genotype information did not appear to compromise the effectiveness or safety of LAI CAB+RPV [22]. Baseline genotype data were less accessible for migrants, reflecting real‐world constraints such as prior diagnosis or care in different countries. This lack of data restricts the assessment of baseline virological risk and the emergence of resistance. Furthermore, previous VF history was missing for 17% of migrants, meaning nearly one in five initiated LAI CAB+RPV without documented ART history. Despite this, migrants performed as well as native‐born participants regarding VF. In Spain, universal ART provision, social coverage and the integration of HIV care within specialist units likely contribute to high effectiveness once patients are engaged. Additionally, the initiation of LAI CAB+RPV in routine practice is typically based on clinical selection, which may have enriched our cohort with individuals possessing stable suppression and established follow‐up, regardless of origin. Importantly, these findings do not imply an absence of structural vulnerability. Key socioeconomic and administrative determinants—such as legal status, housing, employment constraints or health literacy—were not systematically captured in the RELATIVITY cohort.

In our study, migrants experiencing VF had higher baseline BMI, higher HIV viral load at diagnosis and a higher frequency of viral blips in the 5 years preceding the switch. This is consistent with evidence linking non‐sustained viral suppression to pre‐existing factors like high pre‐ART viral load and incomplete suppression on oral therapy [23]. While we could not identify specific socioeconomic predictors for VF due to data limitations, other studies have identified younger age and unemployment as significant risks for migrants [24].

Emergent INSTI drug resistance was prevalent among those experiencing VF, as reported elsewhere [25]. Meta‐analyses show that switching to LAI CAB+RPV carries a non‐significant increased risk of treatment‐emergent RAMs compared with INSTI‐based 2‐ or 3‐drug oral regimens [26]. While most patients failing LAI regimens can be successfully re‐suppressed using single‐tablet oral regimens [27], our observed re‐suppression rate was lower than expected, likely due to short follow‐up; future analysis should re‐evaluate this.

Migrant populations may experience poorer adherence to the HIV care process, affecting adherence, retention and virological response [28]. Access to ART remains inconsistent for migrating populations globally, leading to inequities in care and sometimes restricted access for undocumented migrants [29]. Yet, migrants often face more barriers to retention than to initial treatment access. A review of the HIV care cascade found that 68% of migrants reported retention barriers compared to 30% for linkage to care; two‐thirds of these barriers were individual‐level issues like unfulfilled basic needs (housing, financial stability, work commitments or mental health) that increase disengagement risks [30].

While ART discontinuation is generally higher in migrants across various oral regimens [5], our cohort's adherence—measured as percentage of days covered—was similar regardless of migrant status. This indicates that once initiated, migrants adhered well to the injection schedule.

Nevertheless, discontinuations due to side effects were twice as high in migrants as in Spanish‐born citizens. To our knowledge, this has not been reported elsewhere. Discontinuations due to local side effects were significantly higher, particularly in the on‐label cohort. As meta‐analyses show CAB+RPV carries a higher risk of adverse event‐related discontinuation than oral regimens like bictegravir/emtricitabine/tenofovir alafenamide or dolutegravir/abacavir/lamivudine [26], tolerability is a paramount consideration when switching regimens. The migrant workforce often sustains longer working hours and more physically demanding jobs, which may increase vulnerability to discontinuation due to local pain. Furthermore, implementation factors—injection technique, staff experience, needle length, gluteal injection practices and clinic workflows—as well as potential genetic factors, may influence side‐effect profiles. While no specific systemic side effect was more frequent in migrants, women appeared disproportionately affected.

Travel history and return visits to countries of origin were not recorded in RELATIVTY but are highly relevant, as travel can lead to virological rebound [31]. For migrants on LAI ART, travel may influence persistence in two ways: LAI ART may mitigate adherence challenges during transit, but the requirement for fixed‐interval injections may increase the risk of missed appointments for frequent travellers. Clinicians should consider tailored strategies—extended clinic hours, oral bridging during travel and standardized pain management—to support persistence.

LAI CAB+RPV has proven effective even among PWH with complex medical needs, social vulnerability, unstable housing, mental illness, substance abuse or a history of non‐adherence [32], including those with detectable viraemia at the time of switch [33, 34]. Therefore, migrants with complex social situations are likely to benefit from this regimen. Moreover, LAI CAB+RPV is cost‐effective in our setting [35] and supports the UNAIDS 95‐95‐95 objectives [36].

Our study has limitations. The ambispective design is susceptible to selection and information biases and variability in historical data quality (particularly early post‐switch adverse events for some participants, historical viral blips or treatment failures and baseline genotype availability), which may limit internal validity, generalizability and the strength of causal inferences. However, all participants were actively followed at cohort inclusion, allowing reliable capture of key outcomes. The short median follow‐up precludes long‐term assessment of durability, rare adverse events and late VFs. Additionally, the low prevalence of non‐B subtypes, particularly A6, constrains the evaluation of subtype‐specific risks. A key limitation is the absence of psychosocial and structural data (including administrative status, housing stability, employment constraints, educational level and migration pathway), which restricts causal interpretation of the association between migrant status and treatment discontinuation, particularly for discontinuations attributed to adverse events. Also, the year of arrival in Spain was not systematically captured across participating centres and could not be analysed, although time since arrival may influence structural vulnerability, engagement in care and treatment persistence. Future updates will aim to incorporate structured socioeconomic data. Finally, while management likely differed between centres, patients were treated according to national and European guidelines, limiting these differences. The study's strengths include its large, nationwide, multicentre design, diverse real‐world population and the use of PSM to adjust for confounders.

Our findings provide crucial real‐world evidence supporting the effectiveness of LAI CAB+RPV in migrant PWH in Spain. They underscore that migrants, as a population often experiencing structural and clinical vulnerabilities, face unique challenges with LAI therapy. Addressing these disparities is essential for clinical optimization and promoting health equity [37], ensuring that all populations, and especially vulnerable groups, benefit equally from therapeutic innovations.

## 5 Conclusions

Migrants comprised almost one‐third of individuals switching to LAI. Adherence to the injection schedule was similar to that of native‐born citizens. Despite having less frequent information on baseline genotype and subtype, VF was rare. However, migrants demonstrated a significantly higher risk of LAI CAB+RPV discontinuation, frequently due to adverse events, both local and systemic. These findings highlight the need for closer monitoring and tailored strategies to optimize persistence with LAI regimens in migrant populations.

## Author Contributions

JL‐G: Conceptualization, investigation, methodology, resources, visualization, writing – original draft, writing – review and editing. MMAR: Conceptualization, investigation, methodology, resources, writing – original draft, writing – review and editing. RCH and OBP: Conceptualization, investigation, resources, writing – original draft, writing – review and editing. RPT: Data curation, formal analysis, methodology, software, visualization, writing – original draft, writing – review and editing. MMA, MTGS, MJGP, AR, MPV, ISC, SFM, AC‐U, CNSF, KAC, MPA, MAG, JT, RMMA, MJV, CMH, EBM, NCC, LEMA, LGP, MAS, MRAV, AJSG, CF, CE, EMFP, SGT, CHT, ME, MM‐F, JELG, ACC, MEPD, CMM, VAG, FAR, HPP, SP, MAGJ, LAA, NRV, PNBG, RCM, MA, MVEM, AJT, ARP, MCO, MMGN, JS, JCG, MMS, BC and OLFB: Investigation, resources, writing – review and editing. JT and LB: Conceptualization, investigation, methodology, resources, project administration, data curation, writing – review and editing.

## Funding

No dedicated external funding was received for this study. The RELATIVITY study and this subanalysis were not industry funded, and prospective data collection was performed within routine clinical practice infrastructure across centres.

## Conflicts of Interest

JL‐G has received payment or honoraria for lectures or educational events from Gilead, Viiv/GSK and Janssen. JLG holds a research grant from Gilead, unrelated to this study. MMAR is supported by a Río Hortega fellowship from Instituto de Salud Carlos III‐Fondo Social Europeo CM24/00234 and received personal fees for lectures from Gilead and ViiV/GSK. OBP has received fees as a speaker in educational programmes sponsored by VIIV, Gilead and MSD; and financial support to attend HIV conferences from Gilead and VIIV. MM has received funding as a speaker for events sponsored by Gilead Sciences and ViiV Healthcare and funding for attending conferences from Gilead Sciences. MT has received financial compensation for delivering lectures sponsored by the following pharmaceutical companies: Gilead Sciences, Johnson & Johnson Innovative Medicine and ViiV Healthcare. MJGP has received payment or honoraria for lectures or educational events and advisories from Gilead, Viiv/GSK, Janssen and MSD. MPV has received payment or honoraria for lectures or educational events from Gilead and Viiv/GSK. AC‐U reports grants and personal fees from ViiV Healthcare and Gilead Sciences, personal fees from Johnson & Johnson and Merck, and support for attending meetings and/or travel from ViiV Healthcare, Gilead Sciences, Johnson & Johnson and Merck, unrelated to the submitted work. CNSF has received financial compensation for delivering lectures sponsored by Gilead Sciences. KAC has received payment or honoraria for lectures or educational events from Gilead, Viiv/GSK, Johnson & Johnson, Pfizer and Tilotts, and a research grant from Gilead, unrelated to this study. MAG has received payment or honoraria for lectures or educational events from Gilead, unrelated to this study. JT has received financial compensation for lectures, consultancies and educational activities, as well as research funding from Gilead Sciences, Janssen‐Cilag, Merck Sharp & Dome and ViiV Healthcare. RMMA has received payment or honoraria for lectures or educational events from Gilead, Viiv/GSK, Janssen, Pfizer, Shionogi and Tilotts. MJV has received payment or honoraria for lectures or educational events from AbbVie, Gilead Sciences and ViiV Healthcare, and has sporadically attended congresses funded by Gilead, ViiV Healthcare and Janssen Cilag. CMH has received financial compensation for lectures or educational events sponsored by Gilead Sciences, Johnson & Johnson and ViiV Healthcare. EBM has received lecture fees from Gilead, Janssen, ViiV and MSD, and research grants from Gilead and Janssen. NC‐C has received support for attending meetings and congresses and honoraria as a speaker from Gilead, ViiV Healthcare, Janssen and MSD. CF has received payment or honoraria for lectures or educational events from Gilead, Viiv/GSK, Janssen and MSD. EMFP has received payment for educational events from Gilead, ViiV and Johnson & Johnson. AL‐D has received financial support to attend educational events from Gilead. JELG has consulted for, and received honoraria for speaking, engagements and conference support from Gilead Sciences, Janssen Cilag, Merck Sharp & Dohme, ViiV Healthcare, Pfizer and GSK. FAR has received honoraria as an attendant or speaker in scientific meetings from Johnson & Johnson, Gilead and ViiV. SP has received payment or honoraria for lectures or educational events from Janssen and ViiV. PNBG has received payment or honoraria for lectures or educational events from Gilead and Viiv/GSK. MVEM has received payment for lectures or educational events from Gilead, ViiV and Janssen. ARP has received payment or honoraria for lectures or educational events from Gilead, ViiV/GSK, Janssen, Pfizer and MSD. ARP holds research grants from Gilead and ViiV/GSK, unrelated to this study. MMGN has received payment or honoraria for lectures or educational events from Gilead, Viiv/GSK and Janssen. JS has received honoraria as an attendant or speaker in scientific meetings from Johnson & Johnson, Gilead and ViiV. OLFB has received payment or honoraria for lectures or educational events from Gilead, ViiV and MSD. Likewise, he has received support from the same companies to attend conferences or scientific meetings. JT has received lecture fees from ViiV, Janssen and Gilead; these activities are unrelated to the submitted work. LB‐M reports grants and personal fees from ViiV Healthcare, personal fees from Johnson & Johnson, and support for attending meetings and/or travel from Johnson & Johnson and Merck, unrelated to the submitted work. RCH, RPT, AR, ISC, SFM, MPA, LEMA, LGP, MAS, MRAV, AJSG, CE, SGT, CHT, ME, MM‐F, ACC, MEPD, CMM, VAG, MAGJ, LAA, NRV, RCM, MA, HPP, AJT, MCO, JCG, MMS and BC declare no conflicts of interest.

## Supporting information




**Supporting File 1**: Countries of origin
**Supporting Table 1**: Country of origin of migrants living with HIV starting cabotegravir plus rilpivirine long‐acting injectable in the RELATIVITY cohort, Spain
**Supporting File 2**: Cox proportional hazards models assessing the risk of treatment discontinuation by reason in the overall population.
**Supporting Table 2**: Cox proportional hazards models assessing the risk of treatment discontinuation by reason in the RELATIVITY cohort, Spain
**Supporting File 3**: Propensity score matching analysis
**Supporting Table 3.1**: Baseline characteristics for migrant and Spanish‐born individuals switching to cabotegravir plus rilpivirine long‐acting injectable in the propensity score‐matched subgroup of the RELATIVITY cohort, Spain
**Supporting Table 3.2**: Genotype resistance patterns, specific mutations and HIV‐1 subtypes for migrant and Spanish‐born individuals switching to cabotegravir plus rilpivirine long‐acting injectable in the propensity score‐matched subgroup of the RELATIVITY cohort, Spain
**Supporting Table 3.3**: Persistence, adherence, discontinuation and adverse effects in migrant and Spanish‐born individuals switching to cabotegravir plus rilpivirine long‐acting injectable in the propensity score‐matched subgroup of the RELATIVITY cohort, Spain
**Supporting Figure 3**: (A) Virological failure (VF), (B) systemic adverse events, (C) local injection site reactions, (D) and other reasons for discontinuations for migrants and Spanish‐born individuals living with HIV starting LAI CAB+RPV in a PSM‐adjusted subgroup of the RELATIVITY cohort. HR displayed in the figure are for migrants.
**Supporting Table 3.4**: Cox proportional hazards models assessing the risk of treatment discontinuation by reason in the propensity score‐matched subgroup of the RELATIVITY cohort, Spain.
**Supporting File 4**: On‐label analysis
**Supporting Table 4.1**: Baseline characteristics for migrant and Spanish‐born individuals switching to cabotegravir plus rilpivirine long‐acting injectable in the on‐label subgroup of the RELATIVITY cohort, Spain
**Supporting Table 4.2**: Genotype resistance patterns, specific mutations and HIV‐1 subtypes for migrant and Spanish‐born individuals switching to cabotegravir plus rilpivirine long‐acting injectable in the on‐label subgroup of the RELATIVITY cohort, Spain
**Supporting Table 4.3**: Persistence, adherence, discontinuation and adverse effects for migrant and Spanish‐born individuals switching to cabotegravir plus rilpivirine long‐acting injectable in the on‐label subgroup of the RELATIVITY cohort, Spain
**Supporting Figure 4**: (A) Virological failure (VF), (B) systemic adverse events, (C) local injection site reactions or (D) other reasons for discontinuations in migrants and Spanish‐born individuals living with HIV and switching to LAI CAB+RPV under on‐label conditions in the RELATIVITY cohort.
**Supporting Table 4.4**: Cox proportional hazards models assessing the risk of treatment discontinuation by reason in the on‐label subgroup of the RELATIVITY cohort, Spain.

## Data Availability

The data that support the findings of this study are available on request from the corresponding author. The data are not publicly available due to privacy or ethical restrictions.
