# Supplementary material for: Effectiveness and Persistence of Long‐Acting Injectable Cabotegravir and Rilpivirine in Migrant Individuals Living With HIV in Spain: Substudy of the RELATIVITY Cohort
Source: J Int AIDS Soc. 2026 Apr 21;29(4):e70106. doi: 10.1002/jia2.70106 (PMC13097113; doi:10.1002/jia2.70106)
Supplement: Supplementary file 1 — Supporting File 1: Countries of origin Supporting Table 1: Country of origin of migrants living with HIV starting cabotegravir plus rilpivirine long‐acting injectable in the RELATIVITY cohort, Spain Supporting File 2: Cox proportional hazards models assessing the risk of treatment discontinuation by reason in the overall population. Supporting Table 2: Cox proportional hazards models assessing the risk of treatment discontinuation by reason in the RELATIVITY cohort, Spain Supporting File 3: Propensity score matching analysis Supporting Table 3.1: Baseline characteristics for migrant and Spanish‐born individuals switching to cabotegravir plus rilpivirine long‐acting injectable in the propensity score‐matched subgroup of the RELATIVITY cohort, Spain Supporting Table 3.2: Genotype resistance patterns, specific mutations and HIV‐1 subtypes for migrant and Spanish‐born individuals switching to cabotegravir plus rilpivirine long‐acting injectable in the propensity score‐matched subgroup of the RELATIVITY cohort, Spain Supporting Table 3.3: Persistence, adherence, discontinuation and adverse effects in migrant and Spanish‐born individuals switching to cabotegravir plus rilpivirine long‐acting injectable in the propensity score‐matched subgroup of the RELATIVITY cohort, Spain Supporting Figure 3: (A) Virological failure (VF), (B) systemic adverse events, (C) local injection site reactions, (D) and other reasons for discontinuations for migrants and Spanish‐born individuals living with HIV starting LAI CAB+RPV in a PSM‐adjusted subgroup of the RELATIVITY cohort. HR displayed in the figure are for migrants. Supporting Table 3.4: Cox proportional hazards models assessing the risk of treatment discontinuation by reason in the propensity score‐matched subgroup of the RELATIVITY cohort, Spain. Supporting File 4: On‐label analysis Supporting Table 4.1: Baseline characteristics for migrant and Spanish‐born individuals switching to cabotegravir plus rilpivirine long‐acting injectabl [file JIA2-29-e70106-s001.docx]

# **Supplementary file 1. Countries of origin**

**Table S1.** Country of origin of migrants living with HIV and switching to long-acting injectable cabotegravir plus rilpivirine in the RELATIVITY cohort, Spain.

| **Country** | **n** | **%** |  | **Country** | **n** | **%** |
| --- | --- | --- | --- | --- | --- | --- |
| Colombia | 167 | 17.6% |  | Costa Rica | 2 | 0.2% |
| Venezuela | 148 | 15.6% |  | Croatia | 2 | 0.2% |
| Peru | 71 | 7.5% |  | Lithuania | 2 | 0.2% |
| Argentina | 42 | 4.4% |  | Netherlands | 2 | 0.2% |
| Cuba | 37 | 3.9% |  | The Philippines | 2 | 0.2% |
| Brazil | 36 | 3.8% |  | Switzerland | 2 | 0.2% |
| Ecuador | 36 | 3.8% |  | Algeria | 1 | 0.1% |
| Dominican Republic | 31 | 3.3% |  | Australia | 1 | 0.1% |
| Romania | 23 | 2.4% |  | Austria | 1 | 0.1% |
| Paraguay | 18 | 1.9% |  | Bulgaria | 1 | 0.1% |
| Honduras | 17 | 1.8% |  | Burkina Faso | 1 | 0.1% |
| Italy | 15 | 1.6% |  | Cape Verde | 1 | 0.1% |
| Morocco | 15 | 1.6% |  | China | 1 | 0.1% |
| Equatorial Guinea | 12 | 1.3% |  | Congo | 1 | 0.1% |
| Mexico | 11 | 1.2% |  | Czech Republic | 1 | 0.1% |
| France | 10 | 1.1% |  | Finland | 1 | 0.1% |
| Portugal | 10 | 1.1% |  | The Gambia | 1 | 0.1% |
| El Salvador | 9 | 0.9% |  | Guatemala | 1 | 0.1% |
| Chile | 8 | 0.8% |  | Haiti | 1 | 0.1% |
| Bolivia | 7 | 0.7% |  | India | 1 | 0.1% |
| Nicaragua | 7 | 0.7% |  | Indonesia | 1 | 0.1% |
| Nigeria | 7 | 0.7% |  | Iran | 1 | 0.1% |
| Russia | 5 | 0.5% |  | Kenya | 1 | 0.1% |
| Senegal | 5 | 0.5% |  | Moldova | 1 | 0.1% |
| United Kingdom | 5 | 0.5% |  | Mozambique | 1 | 0.1% |
| Angola | 4 | 0.4% |  | Panama | 1 | 0.1% |
| Cameroon | 4 | 0.4% |  | Slovakia | 1 | 0.1% |
| USA | 4 | 0.4% |  | Tajikistan | 1 | 0.1% |
| Uruguay | 4 | 0.4% |  | Uganda | 1 | 0.1% |
| Germany | 3 | 0.3% |  | Uzbekistan | 1 | 0.1% |
| Guinea | 3 | 0.3% |  | Vatican City | 1 | 0.1% |
| Poland | 3 | 0.3% |  | Unknown | 132 | 13.9% |
| Ukraine | 3 | 0.3% |  | **TOTAL** | **951** | **100.0%** |
| Belgium | 2 | 0.2% |  |  |  |  |

# **Supplementary file 2. Multivariable Cox proportional hazards models assessing the risk of treatment discontinuation by reason in the overall population.**

**Table S2.** Multivariable Cox proportional hazards models assessing the risk of treatment discontinuation by reason in the RELATIVITY cohort, Spain.

| Model/ Variable | aHR (95% CI) | p-value |
| --- | --- | --- |
| Model 1 - Systemic adverse events (n = 2946; events = 27; C-index = 0.75) | | |
| Age at baseline (per year) | 1.05 (1.01–1.09) | 0.015 |
| Gender: woman vs man | 2.63 (1.16–5.88) | 0.021 |
| Migrant vs Spanish-born | 3.33 (1.45–7.59) | 0.005 |
|  |  |  |
| Model 2 – Local injection site reaction (n = 2946; events = 41; C-index = 0.64) | | |
| Migrant vs Spanish-born | 2.63 (1.33–5.26) | 0.005 |
| Hypertension | 2.48 (1.07–5.71) | 0.034 |
|  |  |  |
| Model 3 – Virological failure (n = 2946; events = 19; C-index = 0.62) | | |
| Migrant vs Spanish-born | 2.16 (0.89–5.22) | 0.079 |
|  |  |  |

# **Supplementary file 3. Propensity score matching analysis**

**Table S3.1.** Baseline characteristics for migrant and Spanish-born individuals switching to cabotegravir plus rilpivirine long-acting injectable in the propensity score-matched subgroup of the RELATIVITY cohort, Spain.

|  | **Migrants**  **(N = 947)** | **Spanish-born**  **(N = 947)** | | **OR (95% CI)** | | **p-value** | |  |
| --- | --- | --- | --- | --- | --- | --- | --- | --- |
| Age, years, median (IQR) | 39.8 (33.0, 48.0) | 41.0 (34.3, 49.0) | | NA | | **0.007** | |  |
| Gender |  |  | |  | |  | |  |
| Cisgender woman | 172/947 (18.2%) | 165/947 (17.4%) | | 1.05 (0.83-1.34) | | 0.72 | |  |
| Cisgender man | 763/947 (80.6%) | 777/947 (82%) | | 0.91 (0.71-1.15) | | 0.44 | |  |
| Transgender woman | 12/947 (1.3%) | 5/947 (0.5%) | | 2.42 (0.79-8.79) | | 0.14 | |  |
| BMI, kg/m², median (IQR) | 25.1 (22.1, 27.8) | 24.4 (21.7, 27.4) | | NA | | **0.021** | |  |
| HIV viral load at HIV diagnosis, copies/mL, median (IQR) | 39632.0 (6559.5, 141190.0) | 41900.0 (8380.0, 180000.0) | | NA | | 0.24 | |  |
| Months from diagnosis to first ART initiation, median (IQR) | 1.0 (0.2, 9.0) | 2.0 (0.0, 12.0) | | NA | | 0.26 | |  |
| Years on ART from treatment initiation to start of CAB/RPV, median (IQR) | 7.0 (4.0, 12.0) | 9.0 (5.0, 13.0) | | NA | | **<0.001** | |  |
| CD4 nadir, cells/mm³, median (IQR) | 326.0 (199.0, 496.0) | 389.5 (232.2, 552.0) | | NA | | **<0.001** | |  |
| Baseline CD4, cells/mm³, median (IQR) | 741.0 (548.0, 935.5) | 826.5 (643.0, 1076.8) | | NA | | **<0.001** | |  |
| Drug use |  |  | |  | |  | |  |
| Any drug | 236/863 (27.3%) | 333/885 (37.6%) | | 0.62 (0.51-0.77) | | **<0.001** | |  |
| Smoking | 182/947 (19.2%) | 331/947 (35%) | | 0.44 (0.36-0.55) | | **<0.001** | |  |
| Alcohol | 104/947 (11%) | 85/947 (9%) | | 1.25 (0.92-1.71) | | 0.17 | |  |
| Chemsex | 40/947 (4.2%) | 44/947 (4.6%) | | 0.91 (0.57-1.44) | | 0.74 | |  |
| Other recreational drug use | 54/947 (5.7%) | 58/947 (6.1%) | | 0.93 (0.62-1.38) | | 0.77 | |  |
| HIV transmission route |  |  | |  | |  | |  |
| Unprotected sex (GBMSM) | 632/947 (66.7%) | 595/947 (62.8%) | | 1.19 (0.98-1.44) | | 0.083 | |  |
| Unprotected sex (heterosexual) | 191/947 (20.2%) | 167/947 (17.6%) | | 1.18 (0.93-1.5) | | 0.18 | |  |
| Shared injection equipment | 9/947 (1%) | 43/947 (4.5%) | | 0.2 (0.09-0.42) | | <0.001 | |  |
| Vertical | 6/947 (0.6%) | 20/947 (2.1%) | | 0.3 (0.1-0.77) | | 0.009 | |  |
| Other | 12/947 (1.3%) | 13/947 (1.4%) | | 0.92 (0.38-2.2) | | 1.00 | |  |
| NA | 97/947 (10.2%) | 109/947 (11.5%) | | 0.88 (0.65-1.18) | | 0.42 | |  |
| Prior AIDS diagnosis |  |  | |  | |  | |  |
| Yes | 94/947 (9.9%) | 85/947 (9%) | | 1.12 (0.81-1.54) | | 0.53 | |  |
| No | 790/947 (83.4%) | 795/947 (83.9%) | | 0.96 (0.75-1.24) | | 0.80 | |  |
| NA | 63/947 (6.7%) | 67/947 (7.1%) | | 0.94 (0.64-1.36) | | 0.79 | |  |
| Previous VF to any ART regimen | |  | |  | |  | |  |
| Yes | 19/947 (2%) | 18/947 (1.9%) | | 1.06 (0.52-2.15) | | 1.00 | |  |
| No | 767/947 (81%) | 777/947 (82%) | | 0.93 (0.73-1.18) | | 0.59 | |  |
| NA | 161/947 (17%) | 152/947 (16.1%) | | 1.07 (0.83-1.38) | | 0.62 | |  |
| Third drug at the time of VF | | |  | |  | |  | |
| INSTI | 8/18 (44.4%) | 1/12 (8.3%) | | 8.8 (0.85-424.13) | | **0.049** | |  |
| NNRTI | 4/18 (22.2%) | 5/12 (41.7%) | | 0.4 (0.06-2.61) | | 0.42 | |  |
| PI | 6/18 (33.3%) | 6/12 (50%) | | 0.5 (0.09-2.85) | | 0.46 | |  |
| Number of blips in the 5 years prior to CAB+RPV treatment | | |  | |  | |  | |
| 0 | 667/829 (80.5%) | 719/854 (84.2%) | | 0.77 (0.6-1) | | 0.047 | |  |
| 1 | 97/829 (11.7%) | 85/854 (10%) | | 1.2 (0.87-1.65) | | 0.27 | |  |
| 2 | 30/829 (3.6%) | 23/854 (2.7%) | | 1.36 (0.75-2.47) | | 0.33 | |  |
| 3 | 14/829 (1.7%) | 11/854 (1.3%) | | 1.32 (0.55-3.22) | | 0.55 | |  |
| ≥ 4 | 21/829 (2.5%) | 16/854 (1.9%) | | 1.36 (0.67-2.81) | | 0.41 | |  |
| Detailed history of ART adherence and virological failures (yes) | 372/452 (82.3%) | 390/436 (89.4%) | | 0.55 (0.36-0.82) | | **0.003** | |  |
| Third agent of ART prior to inclusion |  |  | |  | |  | |  |
| INSTI | 761/947 (80.4%) | 725/947 (76.6%) | | 1.25 (1-1.57) | | 0.050 | |  |
| NNRTI | 88/947 (9.3%) | 93/947 (9.8%) | | 0.94 (0.68-1.29) | | 0.76 | |  |
| PI | 39/947 (4.1%) | 51/947 (5.4%) | | 0.75 (0.48-1.18) | | 0.24 | |  |
| Others | 18/947 (1.9%) | 31/947 (3.3%) | | 0.57 (0.3-1.06) | | 0.081 | |  |
| NA | 41/947 (4.3%) | 47/947 (5%) | | 0.87 (0.55-1.36) | | 0.59 | |  |
| Reason for switching |  |  | |  | |  | |  |
| Toxicity | 11/947 (1.2%) | 16/947 (1.7%) | | 0.68 (0.29-1.58) | | 0.44 | |  |
| Drug interaction | 4/947 (0.4%) | 0/947 (0%) | | 9.04 (0.66-Inf) | | 0.13 | |  |
| Simplification | 186/947 (19.6%) | 193/947 (20.4%) | | 0.95 (0.76-1.2) | | 0.73 | |  |
| Comfort/quality of life | 479/947 (50.6%) | 520/947 (54.9%) | | 0.84 (0.7-1.01) | | 0.066 | |  |
| Malabsorption | 9/947 (1%) | 28/947 (3%) | | 0.31 (0.13-0.69) | | **0.002** | |  |
| Swallowing disorders | 3/947 (0.3%) | 5/947 (0.5%) | | 0.6 (0.09-3.09) | | 0.73 | |  |
| Patient request | 360/947 (38%) | 317/947 (33.5%) | | 1.22 (1.01-1.48) | | **0.044** | |  |

ART: antiretroviral treatment; BMI: body mass index; CAB: cabotegravir; CI: confidence interval; GBMSM: gay, bisexual and other men who have sex with men; INSTI: integrase strand transfer inhibitor; IQR: interquartile range; LAI: long-acting injectable; NA: not applicable; NNRTI: non-nucleoside reverse transcriptase inhibitor; NRTI: nucleoside reverse transcriptase inhibitor; OR: odds ratio; PI: protease inhibitor; PID: people who inject drugs; RPV: rilpivirine; VF: virological failure. **In bold:** statistically significant (p<0.05)

**Table S3.2.** Genotype resistance patterns, specific mutations and HIV-1 subtypes for migrant and Spanish-born individuals switching to cabotegravir plus rilpivirine long-acting injectable in the propensity score-matched subgroup of the RELATIVITY cohort, Spain

|  | **Migrants**  **(N = 947)** | **Spanish-born**  **(N = 947)** | **OR (95% CI)** | **p-value** |
| --- | --- | --- | --- | --- |
| Genotype available | 381/947 (40.2%) | 400/947 (42.2%) | 0.92 (0.76-1.11) | 0.40 |
| Subtype |  |  |  |  |
| B | 182/381 (47.8%) | 175/400 (43.8%) | 1.18 (0.88-1.57) | 0.28 |
| A1/A2 | 3/381 (0.8%) | 11/400 (2.8%) | 0.28 (0.05-1.08) | 0.056 |
| F/CRF | 19/381 (5%) | 12/400 (3%) | 1.7 (0.77-3.89) | 0.20 |
| Other | 14/381 (3.7%) | 22/400 (5.5%) | 0.66 (0.31-1.36) | 0.24 |
| NA | 163/381 (42.8%) | 180/400 (45%) | 0.91 (0.68-1.22) | 0.56 |
| Wild type (no mutations) | 254/381 (66.7%) | 283/400 (70.8%) | 0.83 (0.6-1.13) | 0.25 |
| NRTI resistance mutations | 27/381 (7.1%) | 35/400 (8.8%) | 0.8 (0.45-1.38) | 0.43 |
| *184V* | 7/381 (1.8%) | 5/400 (1.2%) | 1.48 (0.4-5.96) | 0.57 |
| *Other nucleoside analog mutations* | 21/381 (5.5%) | 30/400 (7.5%) | 0.72 (0.38-1.33) | 0.31 |
| NNRTI resistance mutations | 38/381 (10%) | 32/400 (8%) | 1.27 (0.76-2.16) | 0.38 |
| *K103N: Yes* | 13/381 (3.4%) | 8/400 (2%) | 1.73 (0.66-4.87) | 0.27 |
| *E138A: Yes* | 5/381 (1.3%) | 0/400 (0%) | 11.7 (1.24-Inf) | **0.013** |
| *Other NNRTI mutations* | 22/381 (5.8%) | 24/400 (6%) | 0.96 (0.5-1.82) | 1.00 |
| Integrase resistance mutations | 8/381 (2.1%) | 4/400 (1%) | 2.12 (0.56-9.71) | 0.25 |
| *L74M/I/F* | 1/381 (0.3%) | 0/400 (0%) | 3.16 (0.2-Inf) | 0.24 |
| *T97A* | 0/381 (0%) | 1/400 (0.2%) | 0.35 (0-5.6) | 0.50 |
| *Other integrase mutations* | 7/381 (1.8%) | 3/400 (0.8%) | 2.48 (0.56-14.94) | 0.21 |
| Total number of detected mutations |  |  |  |  |
| 0 | 321/381 (84.3%) | 346/400 (86.5%) | 0.83 (0.55-1.27) | 0.42 |
| 1 | 50/381 (13.1%) | 39/400 (9.8%) | 1.4 (0.88-2.24) | 0.15 |
| 2 | 9/381 (2.4%) | 15/400 (3.8%) | 0.62 (0.24-1.54) | 0.30 |
| 3 | 1/381 (0.3%) | 0/400 (0%) | 3.16 (0.2-Inf) | 0.24 |

CI: confidence interval; NNRTI: non-nucleoside reverse transcriptase inhibitor; NRTI: nucleoside reverse transcriptase inhibitor; OR: odds ratio. **In bold:** statistically significant (p<0.05)

**Table S3.3.** Persistence, adherence, discontinuation and adverse effects in migrant and Spanish-born individuals switching to cabotegravir plus rilpivirine long-acting injectable in the propensity score-matched subgroup of the RELATIVITY cohort, Spain

|  | **Migrants**  **(N = 947)** | **Spanish-born**  **(N = 947)** | **OR (95% CI)** | **p-value** |
| --- | --- | --- | --- | --- |
| Months of follow-up, median (IQR) | 12.4 (7.0, 18.3) | 15.1 (10.1, 19.5) | NA | **<0.001** |
| Therapeutic adherence, percentage of days covered | |  |  |  |
| <90%  90-99.9% | 13/915 (1.4%)  137/915 (15.0 %) | 19/915 (2.1%)  134/915 (14.6 %) | 0.68 (0.31-1.46)  1.03 (0.80–1.33) | 0.37  0.81 |
| 100% | 765/915 (83.6%) | 762/915 (83.3%) | 1.02 (0.79-1.32) | 0.90 |
| Viral load at follow-up |  |  |  |  |
| <50 copies/mL at month 7 | 376/387 (97.2%) | 396/407 (97.3%) | 0.95 (0.37-2.45) | 1.00 |
| <50 copies/mL at month 13 | 200/210 (95.2%) | 283/292 (96.9%) | 0.64 (0.22-1.78) | 0.35 |
| Treatment discontinuation | 102/947 (10.8%) | 57/947 (6.0%) | 1.79 (1.31 - 2.44) | **<0.001** |
| Temporary | 17/947 (1.8%) | 9/947 (1%) | 1.91 (0.8-4.87) | 0.17 |
| Definitive | 85/947 (9%) | 48/947 (5.1%) | 1.85 (1.26-2.72) | **0.001** |
| LAI CAB+RPV persistence at 6 months | 704/755 (93.2%) | 814/849 (95.9%) | 0.54 (0.34 - 0.85) | **0.008** |
| LAI CAB+RPV persistence at 12 months | 460/480 (95.8%) | 608/619 (98.2%) | 0.41 (0.18 - 0.92) | **0.026** |
| Months to definitive treatment discontinuation, median (IQR) | 7.0 (3.2, 11.3) | 9.1 (4.3, 11.4) | NA | 0.396 |
| Reasons for permanent treatment discontinuation |  |  |  |  |
| VF | 9/947 (1%) | 3/947 (0.3%) | 3.02 (0.75-17.38) | 0.15 |
| Any adverse event | 32/947 (3.4%) | 14/947 (1.5%) | 2.33 (1.24-4.40) | **0.009** |
| *Local injection-site reaction* | 19/947 (2%) | 9/947 (1%) | 2.13 (0.91-5.38) | 0.085 |
| *Systemic adverse effects* | 13/947 (1.4%) | 5/947 (0.5%) | 2.62 (0.87-9.43) | 0.095 |
| Other reasons for discontinuation | 48/947 (5.1%) | 33/947 (3.5%) | 1.48 (0.92-2.4) | 0.11 |
| *Change of residence or transfer* | 21/947 (2.2%) | 35/947 (3.7%) | 0.59 (0.34 - 1.02) | **0.077** |
| *Logistical issues or poor adherence* | 11/947 (1.2%) | 11/947 (1.2%) | 1.00 (0.43 - 2.32) | **1.00** |
| *Pregnancy or pregnancy intention* | 4/947 (0.4%) | 4/947 (0.4%) | 1.00 (0.25 - 4.01) | 1.00 |
| *Medical decision, patient decision or unspecified issues* | 10/947 (1.1%) | 56/947 (5.9%) | 0.17 (0.09 - 0.33) | **<0.001** |

CI: confidence interval; NA: not applicable; OR: odds ratio; VF: virological failure; VL: viral load. **In bold:** statistically significant (p<0.05)


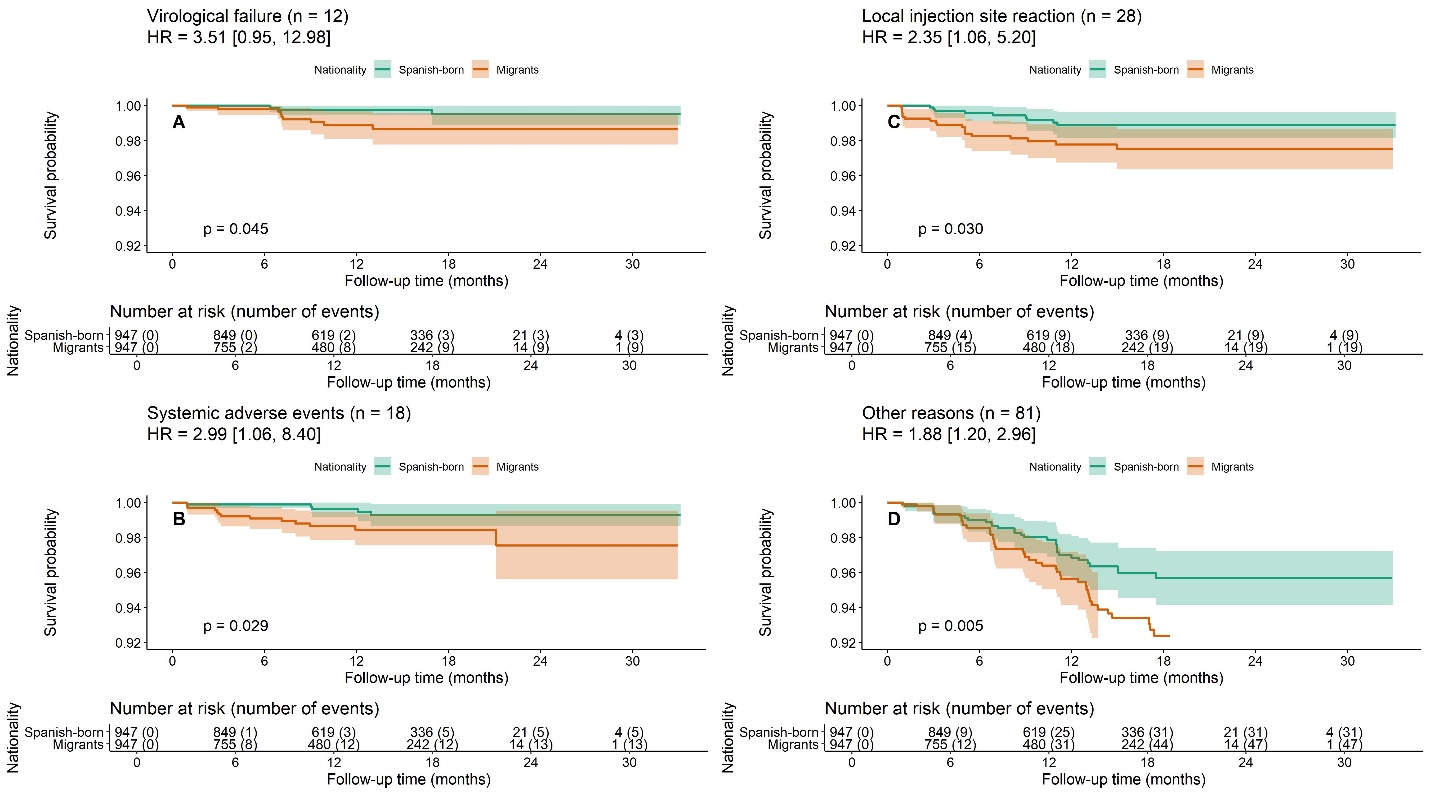


**Figure S3.** (A) Virological failure (VF), (B) systemic adverse events, (C) local injection site reactions, (D) and other reason for discontinuations for migrants and Spanish-born individuals living with HIV starting LAI CAB+RPV in a PSM-adjusted subgroup of the RELATIVITY cohort. HR displayed in the figure are for migrants.

**Table S3.4.** Multivariable Cox proportional hazards models assessing the risk of treatment discontinuation by reason in the propensity score-matched subgroup of the RELATIVITY cohort, Spain.

| Model / Variable | aHR (95% CI) | p-value |
| --- | --- | --- |
| Model 1 – Systemic adverse events (n = 1,894; events = 18; C-index = 0.81) | | |
| Gender: woman vs man | 3.18 (1.16–8.70) | 0.024 |
| Osteoporosis | 3.26 (0.94–11.30) | 0.063 |
| Migrants vs Spanish-born | 2.88 (1.01–8.17) | 0.047 |
|  |  |  |
| Model 2 – Local injection site reaction (n = 1,894; events = 26; C-index = 0.74) | | |
| Gender: woman vs man | 2.84 (1.18–6.85) | 0.020 |
| Dyslipidemia | 2.35 (1.06–5.20) | 0.030 |
| Cerebrovascular disease | 6.63 (0.81–54.58) | 0.079 |
|  |  |  |
| Model 3 – Virological failure (n = 1,894; events = 12; C-index = 0.71) | | |
| Migrants vs Spanish-born | 3.51 (0.95–12.98) | 0.045 |
|  |  |  |

# **Supplementary file 4. On-label analysis**

**Table S4.1.** Baseline characteristics for migrant and Spanish-born individuals switching to cabotegravir plus rilpivirine long-acting injectable in the on-label subgroup of the RELATIVITY cohort, Spain.

|  | **Migrants**  **(N = 894)** | **Spanish-born**  **(N = 2061)** | **OR (95% CI)** | **p-value** |
| --- | --- | --- | --- | --- |
| Age, years, median (IQR) | 39.9 (33.0, 48.0) | 47.6 (40.0, 56.0) | NA | **<0.001** |
| Gender |  |  |  |  |
| Cisgender woman | 164/890 (18.4%) | 266/2056 (12.9%) | 1.52 (1.22-1.89) | **<0.001** |
| Cisgender man | 714/890 (80.2%) | 1784/2056 (86.8%) | 0.62 (0.5-0.77) | **<0.001** |
| Transgender woman | 12/890 (1.3%) | 6/2056 (0.3%) | 4.67 (1.61-15.21) | **0.001** |
| BMI, kg/m², median (IQR) | 25.2 (22.2, 27.8) | 24.6 (21.8, 27.6) | NA | **0.050** |
| HIV viral load at HIV diagnosis, copies/mL, median (IQR) | 37559.0 (6276.5, 140140.2) | 55000.0 (12182.5, 203774.5) | NA | **<0.001** |
| Months from diagnosis to first ART initiation, median (IQR) | 1.0 (0.4, 9.0) | 2.0 (1.0, 21.0) | NA | **<0.001** |
| Years on ART from treatment initiation to start of CAB/RPV, median (IQR) | 7.0 (4.0, 12.0) | 10.0 (6.0, 16.0) | NA | **<0.001** |
| CD4 nadir, cells/mm³, median (IQR) | 322.0 (199.0, 500.0) | 348.0 (199.5, 500.0) | NA | 0.341 |
| Baseline CD4, cells/mm³, median (IQR) | 744.0 (546.0, 940.0) | 806.0 (621.8, 1044.5) | NA | **<0.001** |
| Drug use |  |  |  |  |
| Any drug | 217/813 (26.7%) | 669/1948 (34.3%) | 0.7 (0.58-0.84) | **<0.001** |
| Smoking | 172/894 (19.2%) | 696/2061 (33.8%) | 0.47 (0.38-0.57) | **<0.001** |
| Alcohol | 97/894 (10.9%) | 191/2061 (9.3%) | 1.19 (0.91-1.55) | 0.20 |
| Chemsex | 36/894 (4%) | 72/2061 (3.5%) | 1.16 (0.75-1.77) | 0.52 |
| Other recreational drug use | 49/894 (5.5%) | 103/2061 (5%) | 1.1 (0.76-1.58) | 0.59 |
| HIV transmission route |  |  |  |  |
| Unprotected sex (GBMSM) | 598/894 (66.9%) | 1270/2061 (61.6%) | 1.26 (1.06-1.49) | **0.007** |
| Unprotected sex (heterosexual) | 180/894 (20.1%) | 372/2061 (18%) | 1.14 (0.93-1.4) | 0.18 |
| Shared injection equipment | 7/894 (0.8%) | 163/2061 (7.9%) | 0.09 (0.04-0.19) | **<0.001** |
| Vertical | 5/894 (0.6%) | 15/2061 (0.7%) | 0.77 (0.22-2.23) | 0.81 |
| Other | 11/894 (1.2%) | 27/2061 (1.3%) | 0.94 (0.42-1.97) | 1.00 |
| NA | 93/894 (10.4%) | 214/2061 (10.4%) | 1 (0.77-1.3) | 1.00 |
| Prior AIDS diagnosis |  |  |  |  |
| Yes | 89/894 (10%) | 265/2061 (12.9%) | 0.75 (0.57-0.97) | **0.026** |
| No | 742/894 (83%) | 1672/2061 (81.1%) | 1.14 (0.92-1.41) | 0.23 |
| NA | 63/894 (7%) | 124/2061 (6%) | 1.18 (0.85-1.64) | 0.29 |
| Previous VF to any ART regimen | |  |  |  |
| Yes | 7/894 (0.8%) | 40/2061 (1.9%) | 0.4 (0.15-0.9) | **0.024** |
| No | 726/894 (81.2%) | 1749/2061 (84.9%) | 0.77 (0.62-0.95) | **0.015** |
| NA | 161/894 (18%) | 272/2061 (13.2%) | 1.44 (1.16-1.8) | **0.001** |
| Third drug at the time of VF |  |  |  |  |
| INSTI | — | — | — | — |
| NNRTI | — | — | — | — |
| PI | 6/7 (85.7%) | 24/40 (60.0%) | 4.00 (0.43 - 37.2) | 0.22 |
| Number of blips in the 5 years prior to CAB+RPV treatment | |  |  |  |
| 0 | 628/779 (80.6%) | 1574/1882 (83.6%) | 0.81 (0.65-1.02) | 0.063 |
| 1 | 88/779 (11.3%) | 197/1882 (10.5%) | 1.09 (0.82-1.43) | 0.54 |
| 2 | 29/779 (3.7%) | 56/1882 (3%) | 1.26 (0.77-2.03) | 0.33 |
| 3 | 13/779 (1.7%) | 25/1882 (1.3%) | 1.26 (0.59-2.57) | 0.48 |
| ≥ 4 | 21/779 (2.7%) | 30/1882 (1.6%) | 1.71 (0.92-3.11) | 0.063 |
| Detailed history of ART adherence and VF (yes) | 372/450 (82.7%) | 775/866 (89.5%) | 0.56 (0.4-0.79) | **0.001** |
| Third agent of ART prior to inclusion |  |  |  |  |
| INSTI | 713/894 (79.8%) | 1562/2061 (75.8%) | 1.26 (1.04-1.53) | **0.020** |
| NNRTI | 86/894 (9.6%) | 226/2061 (11%) | 0.86 (0.66-1.13) | 0.30 |
| PI | 38/894 (4.3%) | 120/2061 (5.8%) | 0.72 (0.48-1.05) | 0.091 |
| Others | 16/894 (1.8%) | 63/2061 (3.1%) | 0.58 (0.31-1.02) | 0.062 |
| NA | 41/894 (4.6%) | 90/2061 (4.4%) | 1.05 (0.7-1.55) | 0.77 |
| Reason for switching |  |  |  |  |
| Toxicity | 11/894 (1.2%) | 32/2061 (1.6%) | 0.79 (0.36-1.62) | 0.62 |
| Drug interaction | 4/894 (0.4%) | 6/2061 (0.3%) | 1.54 (0.32-6.51) | 0.50 |
| Simplification | 174/894 (19.5%) | 517/2061 (25.1%) | 0.72 (0.59-0.88) | **0.001** |
| Comfort/quality of life | 455/894 (50.9%) | 1068/2061 (51.8%) | 0.96 (0.82-1.13) | 0.66 |
| Malabsorption | 7/894 (0.8%) | 31/2061 (1.5%) | 0.52 (0.19-1.2) | 0.15 |
| Swallowing disorders | 3/894 (0.3%) | 10/2061 (0.5%) | 0.69 (0.12-2.69) | 0.77 |
| Patient request | 345/894 (38.6%) | 741/2061 (36%) | 1.12 (0.95-1.32) | 0.18 |

ART: antiretroviral treatment; BMI: body mass index; CAB: cabotegravir; CI: confidence interval; GBMSM: gays, bisexuals and other men who have sex with men; INSTI: integrase strand transfer inhibitor; IQR: interquartile range; LAI: long-acting injectable; NA: not applicable; NNRTI: non-nucleoside reverse transcriptase inhibitor; NRTI: nucleoside reverse transcriptase inhibitor; OR: odds ratio; PI: protease inhibitor; PID: people who inject drugs; RPV: rilpivirine; VF: virological failure. **In bold:** statistically significant (p<0.05)

**Table S4.2.** Genotype resistance patterns, specific mutations and HIV-1 subtypes for migrant and Spanish-born individuals switching to cabotegravir plus rilpivirine long-acting injectable in the on-label subgroup of the RELATIVITY cohort, Spain

|  | **Migrants**  **(N = 894)** | **Spanish-born**  **(N = 2061)** | **OR (95% CI)** | **p-value** |
| --- | --- | --- | --- | --- |
| Genotype available | 329/894 (36.8%) | 970/2061 (47.1%) | 0.65 (0.56-0.77) | **<0.001** |
| Subtype |  |  |  |  |
| B | 154/329 (46.8%) | 432/970 (44.5%) | 1.1 (0.85-1.42) | 0.48 |
| A1/A2 | 2/329 (0.6%) | 19/970 (2%) | 0.31 (0.03-1.28) | 0.13 |
| F/CRF | 15/329 (4.6%) | 18/970 (1.9%) | 2.53 (1.17-5.37) | **0.013** |
| Other | 13/329 (4%) | 42/970 (4.3%) | 0.91 (0.44-1.75) | 0.88 |
| NA | 145/329 (44.1%) | 459/970 (47.3%) | 0.88 (0.68-1.14) | 0.34 |
| Wild type (no mutations) | 247/329 (75.1%) | 738/970 (76.1%) | 0.95 (0.7-1.28) | 0.71 |
| NRTI resistance mutations | 17/329 (5.2%) | 64/970 (6.6%) | 0.77 (0.42-1.36) | 0.43 |
| *184V* | 4/329 (1.2%) | 6/970 (0.6%) | 1.98 (0.41-8.39) | 0.28 |
| *Other nucleoside analog mutations* | 12/329 (3.6%) | 59/970 (6.1%) | 0.58 (0.28-1.12) | 0.12 |
| NNRTI resistance mutations | 0/329 (0.0%) | 0/970 (0.0%) | NA | NA |
| Integrase resistance mutations | 0/329 (0.0%) | 0/970 (0.0%) | NA | NA |
| Total number of detected mutations |  |  |  |  |
| 0 | 314/329 (95.4%) | 908/970 (93.6%) | 1.43 (0.79-2.75) | 0.28 |
| 1 | 15/329 (4.6%) | 62/970 (6.4%) | 0.7 (0.36-1.27) | 0.28 |

CI: confidence interval; NA: not applicable; NNRTI: non-nucleoside reverse transcriptase inhibitor; NRTI: nucleoside reverse transcriptase inhibitor; OR: odds ratio. **In bold:** statistically significant (p<0.05)

**Table S4.3.** Persistence, adherence, discontinuation and adverse effects for migrant and Spanish-born individuals switching to cabotegravir plus rilpivirine long-acting injectable in the on-label subgroup of the RELATIVITY cohort, Spain

|  | **Migrants**  **(N = 894)** | **Spanish-born**  **(N = 2061)** | **OR (95% CI)** | **p-value** |
| --- | --- | --- | --- | --- |
| Months of follow-up, median (IQR) | 12.1 (7.0, 17.7) | 14.9 (9.1, 19.2) |  | **<0.001** |
| Therapeutic adherence, percentage of days covered | |  |  |  |
| <90%  90-99.9% | 13/865 (1.5%)  129 / 865 (14.9%) | 35/1990 (1.8%)  270 / 1990 (13.6%) | 0.85 (0.41-1.66)  1.11 (0.89–1.40) | 0.75  0.35 |
| 100% | 723/865 (83.6%) | 1685/1990 (84.7%) | 0.92 (0.74-1.15) | 0.47 |
| Viral load at follow-up |  |  |  |  |
| <50 copies/mL at month 7 | 362/371 (97.6%) | 817/832 (98.2%) | 0.74 (0.3-1.93) | 0.51 |
| <50 copies/mL at month 13 | 190/200 (95%) | 571/585 (97.6%) | 0.47 (0.19-1.19) | 0.092 |
| Treatment discontinuation | 96/894 (10.7%) | 116/2061 (5.6%) | 1.95 (1.47 - 2.58) | **<0.001** |
| Temporary | 13/894 (1.5%) | 13/2061 (0.6%) | 2.32 (0.99-5.47) | **0.033** |
| Definitive | 83/894 (9.3%) | 103/2061 (5%) | 1.95 (1.42-2.65) | **<0.001** |
| LAI CAB+RPV persistence at 6 months | 660/709 (93.1%) | 1744/1812 (96.2%) | 0.48 (0.33 - 0.70) | **<0.001** |
| LAI CAB+RPV persistence at 12 months | 432/450 (96%) | 1278/1300 (98.3%) | 0.43 (0.22 - 0.83) | **0.010** |
| Months to definitive treatment discontinuation, median (IQR) | 6.9 (3.1, 11.1) | 8.8 (4.7, 11.8) | NA | 0.169 |
| Reasons for permanent treatment discontinuation |  |  |  |  |
| VF | 9/894 (1%) | 10/2061 (0.5%) | 2.09 (0.75-5.73) | 0.13 |
| Any adverse event | 32/894 (3.6%) | 36/2061 (1.8%) | 2.04 (1.26-3.32) | **0.004** |
| *Local injection-site reaction* | 19/894 (2.1%) | 22/2061 (1.1%) | 2.01 (1.02-3.92) | **0.038** |
| *Systemic adverse effects* | 13/894 (1.5%) | 14/2061 (0.7%) | 2.16 (0.93-4.97) | 0.056 |
| Other reasons for discontinuation | 46/894 (5.1%) | 61/2061 (3%) | 1.78 (1.18-2.67) | **0.005** |
| *Change of residence or transfer* | 21/894 (2.3%) | 17/2061 (0.8%) | 2.89 (1.52–5.51) | **0.001** |
| *Logistical issues or poor adherence* | 11/894 (1.2%) | 11/2061 (0.5%) | 2.32 (1.00–5.37) | **0.060** |
| *Pregnancy or pregnancy intention* | 4/894 (0.4%) | 2/2061 (0.1%) | 4.63 (0.85–25.31) | 0.072 |
| *Medical decision, patient decision or unspecified issues* | 10/894 (1.1%) | 30/2061 (1.5%) | 0.77 (0.37–1.57) | 0.60 |

CI: confidence interval; NA: not applicable; OR: odds ratio; VF: virological failure; VL: viral load. **In bold:** statistically significant (p<0.05)


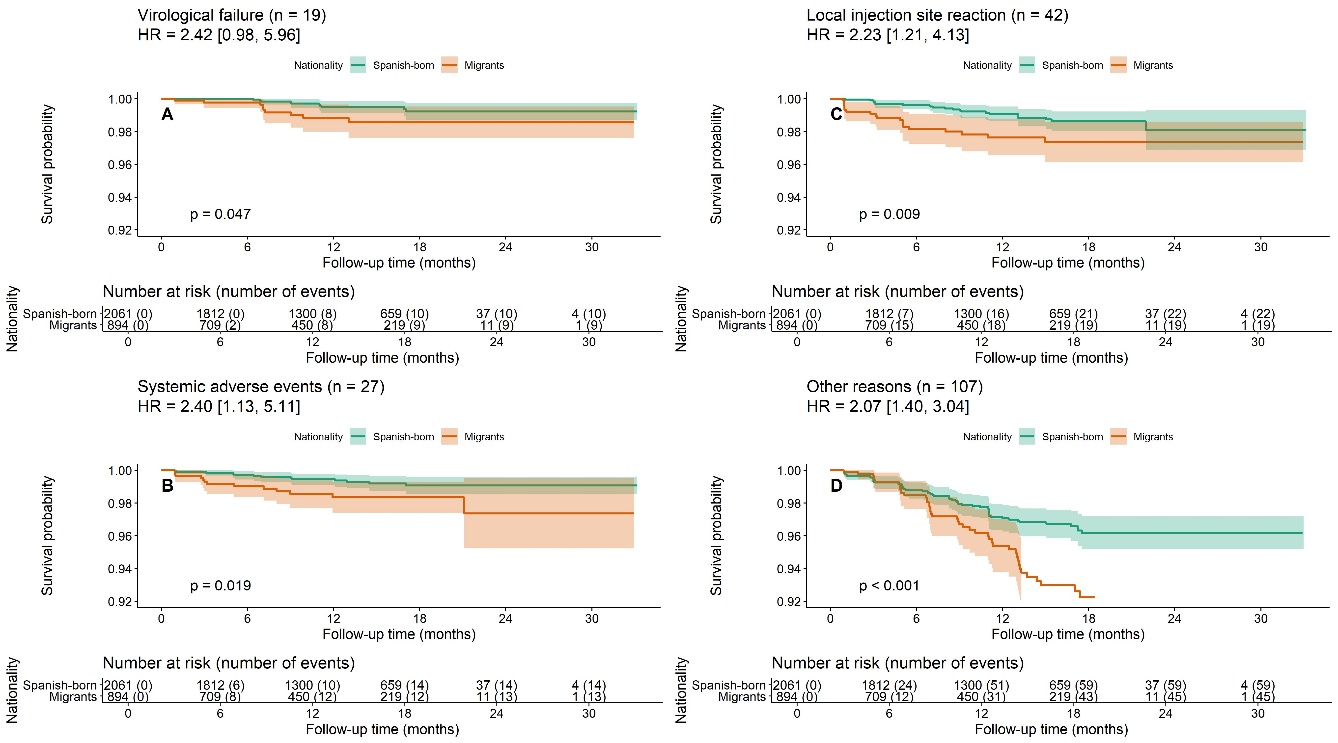


**Figure S4.** (A) Virological failure (VF), (B) systemic adverse events, (C) local injection site reactions, (D) and other reason for discontinuations in migrants and Spanish-born individuals living with HIV and switching to LAI CAB+RPV under on-label conditions in the RELATIVITY cohort. HR displayed in the figure are for migrants

**Table S4.4.** Multivariable Cox proportional hazards models assessing the risk of treatment discontinuation by reason in the on-label subgroup of the RELATIVITY cohort, Spain.

| Model / Variable | aHR (95% CI) | p-value |
| --- | --- | --- |
| Model 1 – Systemic adverse events (n = 2,928; events = 25; C-index = 0.74) | | |
| Age at baseline (per year) | 1.04 (1.01–1.08) | 0.024 |
| Gender woman vs man | 2.73 (1.16–6.25) | 0.017 |
| Migrants vs Spanish-born | 2.72 (1.20–6.20) | 0.017 |
| Chronic Renal Disease | 3.92 (0.87–17.60) | 0.075 |
|  |  |  |
| Model 2 – Local injection site reaction (n = 2,663; events = 34; C-index = 0.66) | | |
| Migrants vs Spanish-born | 2.63 (1.33–5.18) | 0.005 |
| Diabetes mellitus | 2.48 (1.07–5.71) | 0.034 |
| Model 3 – Virological failure (n = 2,928; events = 19; C-index = 0.63) | | |
| Migrants vs Spanish-born | 2.42 (0.98–5.96) | 0.047 |
|  |  |  |
